# Modeling Liver Organogenesis by Recreating Three-Dimensional Collective Cell Migration: A Role for TGFβ Pathway

**DOI:** 10.3389/fbioe.2021.621286

**Published:** 2021-06-15

**Authors:** Ogechi Ogoke, Osama Yousef, Cortney Ott, Allison Kalinousky, Wayne Lin, Claire Shamul, Shatoni Ross, Natesh Parashurama

**Affiliations:** ^1^Department of Chemical and Biological Engineering, University at Buffalo (State University of New York), Buffalo, NY, United States; ^2^Department of Biomedical Engineering, University at Buffalo (State University of New York), Buffalo, NY, United States; ^3^Clinical and Translational Research Center, University at Buffalo (State University of New York), Buffalo, NY, United States

**Keywords:** cell migration, hepatocyte migration, hepatic cords, liver regenerative medicine, cancer metastasis, liver cancer, liver development

## Abstract

Three-dimensional (3D) collective cell migration (CCM) is critical for improving liver cell therapies, eliciting mechanisms of liver disease, and modeling human liver development and organogenesis. Mechanisms of CCM differ in 2D vs. 3D systems, and existing models are limited to 2D or transwell-based systems, suggesting there is a need for improved 3D models of CCM. To recreate liver 3D CCM, we engineered *in vitro* 3D models based upon a morphogenetic transition that occurs during liver organogenesis, which occurs rapidly between E8.5 and E9.5 (mouse). During this morphogenetic transition, 3D CCM exhibits co-migration (multiple cell types), thick-strand interactions with surrounding septum transversum mesenchyme (STM), branching morphogenesis, and 3D interstitial migration. Here, we engineer several 3D *in vitro* culture systems, each of which mimics one of these processes *in vitro*. In mixed spheroids bearing both liver cells and uniquely MRC-5 (fetal lung) fibroblasts, we observed evidence of co-migration, and a significant increase in length and number of liver spheroid protrusions, which was highly sensitive to transforming growth factor beta 1 (TGFβ1) stimulation. In MRC-5-conditioned medium (M-CM) experiments, we observed dose-dependent branching morphogenesis associated with an upregulation of Twist1, which was inhibited by a broad TGFβ inhibitor. In models in which liver spheroids and MRC-5 spheroids were co-cultured, we observed complex strand morphogenesis, whereby thin, linear, 3D liver cell strands attach to the MRC-5 spheroid, anchor and thicken to form permanent and thick anchoring contacts between the two spheroids. In these spheroid co-culture models, we also observed spheroid fusion and strong evidence for interstitial migration. In conclusion, we present several novel cultivation systems that recreate distinct features of liver 3D CCM. These methodologies will greatly improve our molecular, cellular, and tissue-scale understanding of liver organogenesis, liver diseases like cancer, and liver cell therapy, and will also serve as a tool to bridge conventional 2D studies and preclinical *in vivo* studies.

## Introduction

Liver (epithelial) cell migration plays important roles in liver organogenesis, disease, and therapy. During liver organogenesis (E8.5, mouse), hepatic endoderm lining the liver diverticulum (LD) undergoes epithelial to mesenchymal transition (EMT) and initiates three-dimensional (3D) collective cell migration (CCM), leading to formation of the hepatic cords which extend into the surrounding connective tissue [septum transversum mesenchyme (STM)] to form the liver bud (Bort et al., 2006). Later stages of organ development also require liver cell migration. Studies of rat fetal hepatoblast expression indicate that genes associated with 3D CCM, morphogenesis, and extracellular matrix remodeling, are also highly upregulated in fetal hepatoblasts (Petkov et al., 2000). This strongly suggests that interstitial migration plays a role in fetal liver growth. 3D CCM is critical in hepatocellular carcinoma (HCC), and studies demonstrate that local spread and metastasis corresponds to worsened prognosis and increased treatment resistance (Yang et al., 2009). Finally, liver 3D CCM is important for successful liver cell therapy, in which either adult or fetal hepatocytes are employed for acute and chronic liver disease models. During cell therapy, non-invasive imaging has demonstrated that transplanted hepatocytes enter the portal vein, then capillaries within hours, migrate across the liver sinusoids, and through liver tissue (Rajvanshi et al., 1996; Gupta et al., 1999; Koenig et al., 2005). Thus, 3D liver CCM is important for liver development, interstitial migration during fetal liver growth, HCC, and liver cell therapy.

Genetic studies have elicited molecular pathways that drive 3D liver CCM. These studies identify several phases of liver 3D CCM including cell strand (cord) formation, co-migration involving multiple cell types, branching morphogenesis, and interstitial migration. Hepatic cords are required for liver organogenesis. Mouse genetic and explant studies of hepatic cord formation demonstrate that hepatic endoderm co-migrates with endothelial cells (Matsumoto et al., 2001) and STM cells, forms thick cell strands that interact with the mesenchyme, and undergoes branching suggestive of early 3D branching morphogenesis (Watt et al., 2007), eventually forming primitive cell sheets consisting of hepatoblasts (Sosa-Pineda et al., 2000). 3D CCM in the LD occurs in part in response to secreted fibroblast growth factor 2 (FGF2) from the cardiac mesoderm, bone morphogenetic protein (BMP4) from the STM, and endothelial cell interactions (Ledouarin, 1964; Gualdi et al., 1996; Matsumoto et al., 2001; Rossi et al., 2001), and migration-associated transcription factors include Hex (Martinez Barbera et al., 2000), Prox1 (Sosa-Pineda et al., 2000), and Tbx3 (Suzuki et al., 2008). For example, GATA4– embryos do not form the STM, and this leads to a lack of hepatic cord formation and subsequent liver development (Watt et al., 2007; Delgado et al., 2014). Along these lines, conditional knockout of Hlx disrupts liver formation ameliorates necessary mesenchymal-epithelial cell interactions (Hentsch et al., 1996). In hepatocyte growth factor (HGF) (–) mutants without HGF secretion, presumably by the STM, the liver is hypoplastic and exhibits apoptosis (Schmidt et al., 1995; Uehara et al., 1995). Furthermore, in Smad 2/3 (±) double heterozygotes, which mediate (transforming growth factor beta 1) TGFβ1 signaling, the liver is hypoplastic with hepatoblast clustering, and mislocalization of Beta-1 integrin expression (Weinstein et al., 2001). These genetic studies collectively indicate that reciprocal, molecular interactions between hepatoblast and mesenchyme are necessary for 3D liver migration and growth. They also highlight that several phases of liver 3D CCM are important, including co-migration, branching morphogenesis, strand (cord) formation, and interstitial migration.

Current *in vitro* studies of human hepatic migration include 2D and 3D systems. Most commonly utilized are 2D assays of highly migratory HCC cells combined with *in vivo* heterotopic or orthotopic tumor models. Using this approach, scientists have demonstrated various factors that control 2D HCC migration including TGFβ1 (Fransvea et al., 2008; Ng et al., 2013), c-Myc (Zhao et al., 2013), YAP (Fitamant et al., 2015), goosecoid (Xue et al., 2014), actopaxin (Biname et al., 2008), and more recently miRNAs [miRNA-135a (Zeng et al., 2016), miRNA-338-3p (Chen et al., 2017), miRNA-1301 (Yang et al., 2017), miRNA-665 (Hu et al., 2018)]. The limitations of 2D assays are that they cannot model 3D liver CCM, they take place on rigid plastic surfaces which are non-physiological, and that heterotypic cell interactions are difficult to model in these systems.

Recent liver studies have provided some optimism for modeling 3D liver migration, and studies in other epithelial tissue systems demonstrate CCM. It is important to generate 3D CCM, since 3D CCM is known to exhibit a varied collection of migration modes (Friedl and Gilmour, 2009; Wu et al., 2014). Co-cultivation of human umbilical vein endothelial cells (HUVEC), human mesenchymal stem cells (hMSC), and human stem cell-derived hepatic endoderm in 3D organoids demonstrate that heterotypic cell interactions drive *in vitro* liver organoid growth and differentiation (Camp et al., 2017), including via pathways like TNF, JAK/STAT, NF-κB, HIF, and VEGF. However, these studies do not explicitly demonstrate 3D CCM and nor do they demonstrate clear radial migration that occurs during the stage of development between liver diverticulum LD and liver bud. Recently, a promising 3D model of HCC migration, employing LX-2 (hepatic stellate cell line), has demonstrated that mesenchymal components in spheroids affect extracellular matrix protein expression and TGFβ1 expression, collective cell movement, and altered drug sensitivity (Khawar et al., 2018). While these studies also support the importance of supporting mesenchyme, these studies do not investigate the multiple modes of 3D CCM, and they solely identify co-migration as the primary mechanism of 3D CCM. Progress in other (non-liver) tissue systems has demonstrated the complexity of 3D CCM, including breast cancer and head and neck cancer, demonstrating paradoxical results (Shin et al., 2017), and complex modes of 3D CCM (Lehmann et al., 2017). Taken together, *in vitro* human liver 3D CCM models are in development and appear to be lagging behind the development of other *in vitro* systems for epithelial cancers. Thus, mechanisms of liver 3D CCM remain poorly understood because of a lack of models, suggesting that new model systems are needed to broadly address mechanisms of liver 3D CCM.

**FIGURE 1:**
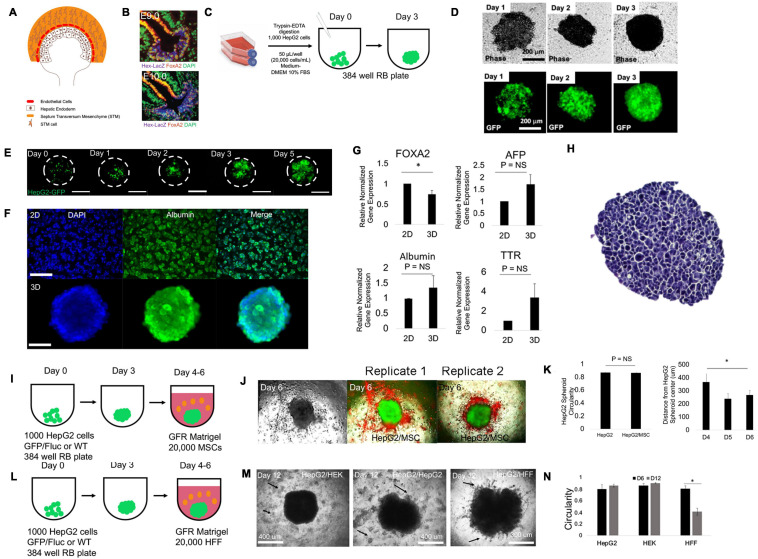
Mesenchymal stem cells (hMSC) induce liver cell migration in 2D models but not in 3D models of the liver diverticulum (LD). **(A)** Schematic of LD during mouse liver development (E8.5). Shown are epithelial hepatic progenitor cells (brown), endothelial precursors (red), and the septum transversum mesenchyme (STM) (orange). At E9.5, hepatic progenitor cells together with endothelial cells, co-migrates and interacts with STM and STM cells, forming hepatic cords. **(B)** The LD identified in the embryological liver (E9.0, top) give rise to hepatic cords migrating (E9.5, bottom) with evidence of co-migration, branching, and interstitial migration, marked by Hex and Foxa2. Figure adopted with permission from Bort et al. (2006). **(C)** Schematic for making spheroid from HepG2 or HepG2-GFP cells. One thousand cells are added in 50 μl and to a 384-well round-bottom non-adherent plate and cultured for 3 days. **(D)** Phase-contrast (top) and corresponding fluorescent images (below) of HepG2-GFP/Fluc from days 1–3 during spheroid formation. Bar = 200 μm. **(E)** Fluorescent images of dilution experiments of HepG2-GFP/HepG2 (1:10) mixed spheroids on days 0–5 during spheroid formation. Bar = 300 μm. **(F)** Fluorescent images of DAPI (nucleus) and albumin in Hep2 monolayer (top) and spheroid (bottom) culture on day 3 of culture. **(G)** qRT-PCR analysis of Foxa2 (*P* = 0.041, *n* = 3), alpha-fetoproteien (AFP), albumin, and transthyretin (TTR) (*P* = NS) in monolayer and day 3 spheroid culture compared with control (*n* = 3). Plotted is mean ± SD. Significance defined as *P* ≤ 0.05. **(H)** Hematoxylin and eosin-stained 10 μm section of HepG2 spheroid in suspension culture illustrates uniform epithelial morphology of 3D tissue. Bar = 200 μm. **(I)** Schematic for modeling the LD. Spheroids are made as in **(G)** and then embedded in 20,000 MSC containing growth factor-free (GFR) MG and cultured for long term. **(J)** Phase (left), double fluorescent (red/green) images (right) of days 4, 5, and 6 LD models, bearing a HepG2-GFP spheroid (green) and MSC (red) in MG. Right columns of images are replicates 1 and 2 with double fluorescent images on days 4, 5, and 6. Bar = 500 μm. **(K)** Bar graph plotting spheroid circularity (left) of HepG2 and HepG2/MSC spheroids (*P* = NS; *n* = 3) and (MSC) distance from HepG2 spheroid on days 4, 5, and 6. days 4 to 6 compared (*P* = 0.00043; *n* = 3). Plotted is mean ± SD. **(L)** Schematic for modeling the LD. HepG2 spheroids are embedded in 20,000 human foreskin fibroblasts (HFF) containing growth factor-free (GFR) MG and cultured for long term. **(M)** Phase-contrast images on day 12 of culture of negative control [HepG2 spheroid embedded with human embryonic kidney (HEK) cells (HepG2/HEK)], a negative control (HepG2/HepG2), and the experimental (HepG2/HFF) condition all at 1:1 ratio. Arrows in the HepG2/HepG2 condition and the HepG2/HEK condition specify the relative location of the HepG2 and HEK cells seeded in the surrounding GFR MG. Arrows in the HFF condition specify the occurrence of co-migration of both HepG2 and HFF cells into the MG. **(N)** Bar graph plotting spheroid circularity in HepG2/HepG2, HepG2/HEK, and HepG2/HFF spheroids. Comparison of HepG2/HFF shown (*P* = 0.000034; *n* = 3). Plotted is mean ± SD. Significance defined as *P* ≤ 0.05. ^∗^ is used to denote significance of experimental data.

**FIGURE 2:**
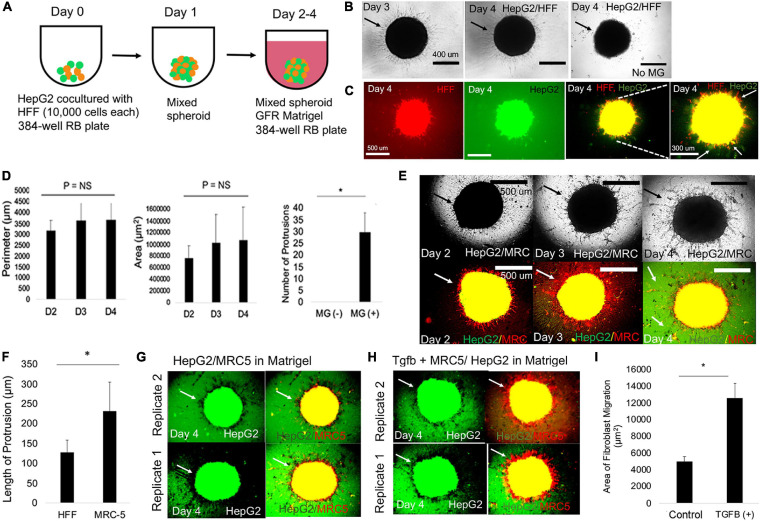
Mixed spheroids with fibroblasts result in 3D livercollective cell migration. **(A)** Schematic for modeling the LD with mixed spheroid containing HepG2 cells and HFF cells cultivated for 1 day followed by embedding in MG from days 2–4. **(B)** Phase-contrast images of HepG2-GFP/HFF-mixed spheroids in MG on days 3 and 4, and day 4 no MG (negative control) condition. Arrows in the HepG2/HFF condition migration of HFF cells into the MG. In the control condition containing no MG, arrows denote lack of migration. **(C)** Fluorescent images of day 4 HepG2-GFP/HFF-mixed spheroids in MG. From left to right: HFF (red) cells, HepG2-GFP (green), HFF/HepG2 (red, green), and HFF/HepG2 (high magnification). White arrows show red, yellow, and green projections. **(D)** Bar graph plotting perimeter (left), area (middle) of day 2 HepG2-GFP/HFF-mixed spheroids over time. *P* = NS (*n* = 3). Right: bar graph plotting number of protrusions in the HepG2/HFF spheroids on day 4 in without MG (MG -) and with MG (MG+) (*P* = 0.022, *n* = 3). Plotted is mean ± SD. Significance defined as *P* ≤ 0.05. **(E)** Phase-contrast (above) and fluorescent images (red, green) below of HepG2-GFP/MRC-5-mixed spheroids in MG on days 2, 3, and 4 and a day 4. Arrow in the picture specifies emergence of finger-like protrusions. **(F)** Bar graph comparing length of protrusion (*P* = 0.0076, *n* = 3) on day 4 between HFF and MRC-5 experimental condition. Plotted is mean ± SD. Significance defined as *P* ≤ 0.05. **(G)** Fluorescent images of day 4 of HepG2-GFP/MRC-5-mixed spheroids in MG. From left to right: HepG2 (green) cells and combined HepG2 (red) and MRC-5 (yellow) images. Replicates 2 (above) and 1 (below) are shown. Arrows show HepG2 and MRC-5 migration. **(H)** Fluorescent images of day 4 of HepG2-GFP/MRC-5-mixed spheroids in MG after treatment with TGFβ1 (20 ng/ml). From left to right: HepG2 (green) cells and combined HepG2 (red) and MRC-5 (yellow) images. Replicates 2 (above) and 1 (below) are shown. Arrows show HepG2 and MRC-5 migration. **(I)** Bar graph comparing area of fibroblast migration in negative control (HepG2-GFP/MRC-5) and (HepG2-GFP/MRC-5 + TGFβ1, 20 ng/ml), *P* = 0.012, *n* = 3. Plotted is mean ± SD. Significance defined as *P* ≤ 0.05. ^∗^ is used to denote significance of experimental data.

**FIGURE 3:**
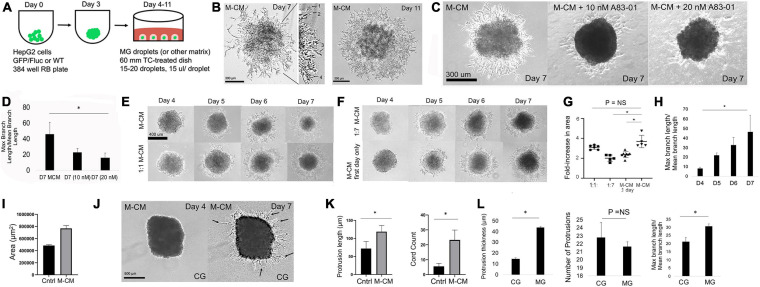
MRC-5-conditioned medium induces 3D collective cellmigration within the *in vitro* liver diverticulum model**. (A)** Schematic for modeling the LD in MG droplets. HepG2 spheroids are embedded in MG droplets (15 μl) with 15–20 droplets per dish and cultured in M-CM medium. **(B)** Phase-contrast images of day 7 (low and high magnification) and day 11 liver spheroid in the MG droplet system cultivated with M-CM. Left: high-magnification view demonstrates various stages/modes of 3D collective migration including the following: (1) filopodia branching, (2) filopodia elongation, (3) cell and nuclear extension (nucleus is visualized), and (4) branches that are interacting. **(C)** Phase-contrast images on day 7 of HepG2 spheroids in MG exposed to M-CM alone, M-CM with A83-01 (10 nM), and A83-01 (20 nM). **(D)** Bar graph analysis comparing day 7 M-CM and MCM + A83-01 (20 nM) (*P* = 0.047, *n* = 3). Plotted is mean ± SD. Significance defined as *P* ≤ 0.05. **(E)** Phase-contrast images of HepG2 spheroids in MG droplet assay in study of M-CM dilution on days 4–7. Conditions tested were M-CM and 1:1 M-CM. **(F)** Phase-contrast images HepG2 spheroids in MG droplet assay in study of M-CM dilution on days 4–7. Conditions tested were 1:7 M-CM and M-CM first-day only. For M-CM first-day only, M-CM was added from days 3 to 4, washed gently, and then switched to M-CM. **(G)** Analysis of MG droplet assay with M-CM. Left, plot of fold change in area across different M-CM dilutions (1:1, 1:7, M-CM 1 day only, and M-CM). Comparison of M-CM with 1:1 condition (*P* = 0.15, *n* = 3 for both conditions), M-CM with 1:7 condition (*P* = 0.0056, *n* = 3 for both conditions), and M-CM with M-CM 1-day-only condition (*P* = 0.019, *n* = 3 for both conditions). **(H)** Analysis of branching of morphogenesis (max branch length/mean branch length) between days 4 and 7 (*P* = 0.047, *n* = 3). Plotted is mean ± SD. Significance defined as *P* ≤ 0.05. **(I)** Bar graph comparing growth area of HepG2 spheroid in MG droplet assay in control (no CM) with M-CM conditions on day 7 of culture (*P* = 0.010, *n* = 3). Plotted is mean ± SD. Significance defined as *P* ≤ 0.05. **(J)** Phase-contrast images on days 4 and 7 of HepG2 spheroid in collagen (CG) droplet and M-CM medium. Arrows specify thin filopodia-like extensions into collagen. **(K)** Bar graph analysis of HepG2 spheroids in CG in control and M-CM conditions comparing protrusion length (*P* = 0.012, *n* = 3) with cord count (*P* = 0.007, *n* = 3). **(L)** Bar graph analysis in CG and MG conditions comparing protrusion thickness (*P* = 0.0000027, *n* = 3), number of protrusion (*P* = NS), and max branch length/mean branch length (*P* = 0.010, *n* = 3). Plotted is mean ± SD. Significance defined as *P* ≤ 0.05. ^∗^ is used to denote significance of experimental data.

**FIGURE 4:**
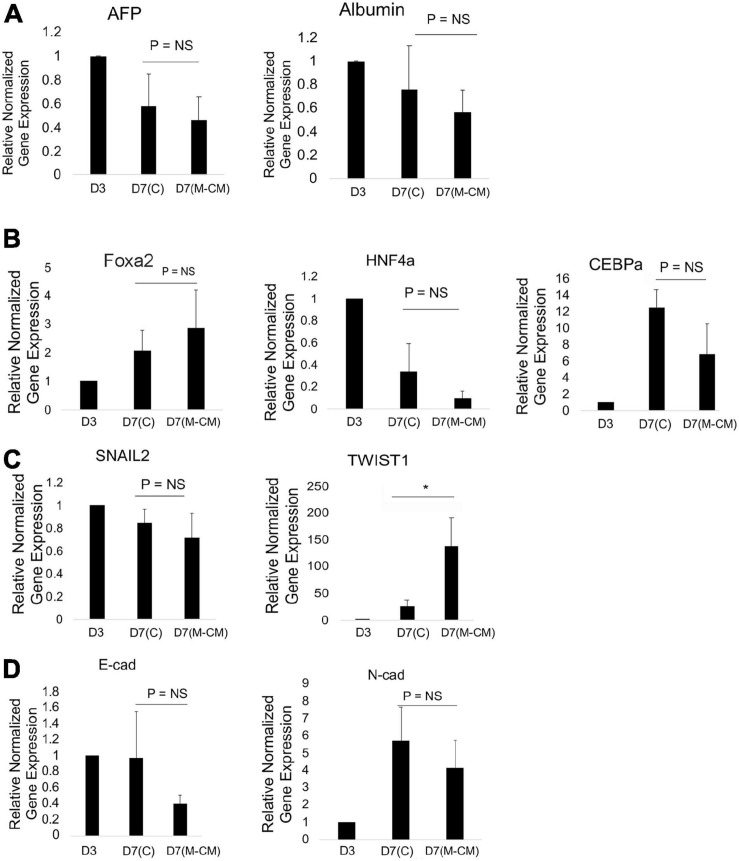
Gene expression analysis of HepG2 spheroids in MG droplets in M-CM. qRT-PCR analysis of HepG2 spheroids on day 3 and after culture in MG droplets with M-CM until day 7. **(A)** Markers associated with liver differentiation, including alpha-fetoprotein (AFP) (*P* = NS, *n* = 3 for day 7 control, *n* = 11 for day 7 M-CM), albumin (Alb) (*P* = NS, *n* = 3 for day 7 control, *n* = 9 for day 7 M-CM) are shown. Plotted is mean ± SD. Significance defined as *P* ≤ 0.05. **(B)** Transcription factors in liver development and maturation including Foxa2 [*P* = NS (*P* = 0.061), *n* = 3 for day 7 control, *n* = 5 for day 7 M-CM], HNF4a [*P* = NS (*P* = 0.25), *n* = 3 for day 7 control, *n* = 10 for day 7 M-CM], CEBPa [*P* = NS, (*P* = 0.103), *n* = 3 for day 7 control, *n* = 3 for day 7 M-CM] are shown. Plotted is mean ± SD. Significance defined as *P* ≤ 0.05.**(C)** Transcription factors associated with epithelial to mesenchymal transition (EMT) including SNAIL 2 (*P* = NS, *n* = 3 for day 7 control, *n* = 3 for day 7 M-CM) and TWIST1 (*P* = 0.026, *n* = 3 for day 7 control, *n* = 4 for day 7 M-CM) were tested. Plotted is mean ± SD. Significance defined as *P* ≤ 0.05. **(D)** Cadherin expression associated with EMT, *E*-cadherin (*E*-Cad) [*P* = NS (*P* = 0.14), *n* = 3 for day 7 control, *n* = 4 for day 7 M-CM] and *N*-cadherin (*N*-Cad) expression (*P* = NS, *n* = 4 for day 7 control, *n* = 4 for day 7 M-CM). Plotted is mean ± SD. Significance defined as *P* ≤ 0.05. ^∗^ is used to denote significance of experimental data.

**FIGURE 5:**
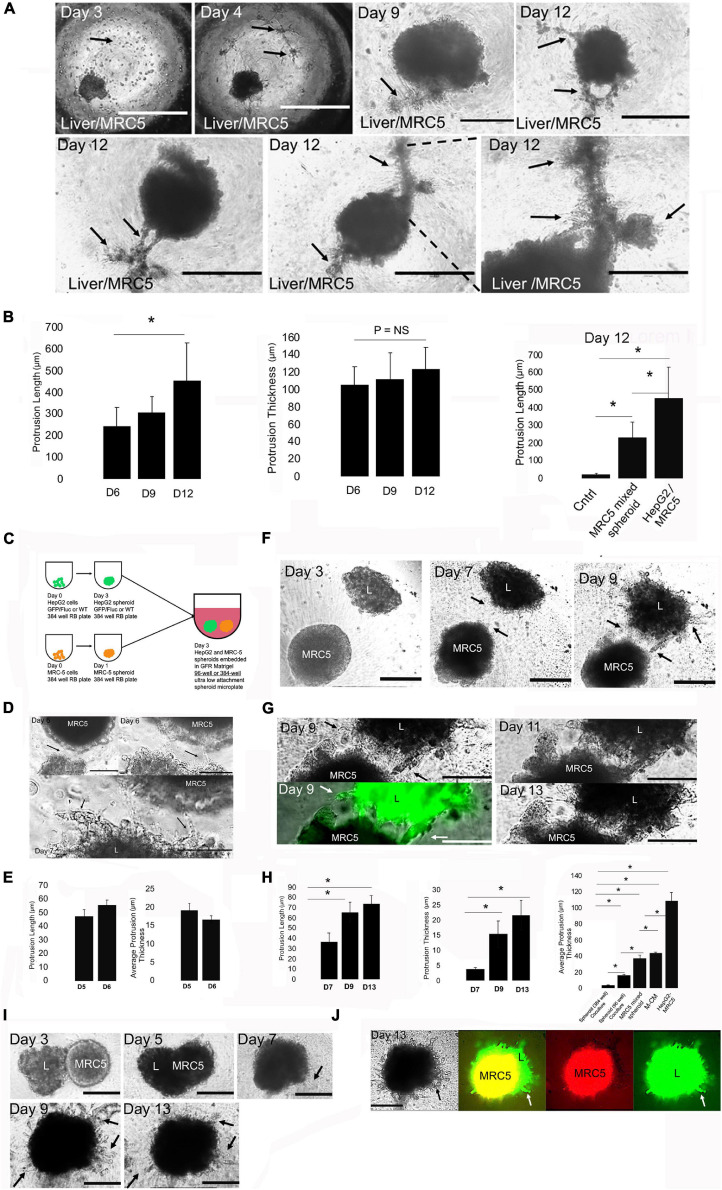
Engineering fibroblast density and format for increased 3D collective cell migration. **(A)** Phase-contrast microscopy images of HepG2 spheroids cultured in MG bearing high density (30,000 cells) of MRC-5 cells. Top row, left to right: days 3, 4, 9, and 12. Day 3: MRC-5 cells initially after seeding (arrow). Bar = 1,000 μm. Day 4: MRC-5 cells spreading and interconnecting (arrows). Bar = 1,000 μm. Day 9: Thick hepatic cord (arrows). Bar = 400 μm. Day 12: multiple, thick hepatic cords. Bar = 400 μm. Bottom row, left to right: each image in a separate experimental replicate on day 12 demonstrating thick hepatic cord formation (arrows). Bar = 400 μm. **(B)** Bar graph analysis of HepG2 spheroids cultured in MG bearing high density (30,000 cells) of MRC-5 cells. Left to right, left: protrusion length on days 6, 9, and 12 shown, days 9 and 12 compared, *P* = 0.05, *n* = 3. Middle: protrusion thickness on days 6, 9, and 12 shown, days 9 and 12 compared, *P* = NS, *n* = 3. Right: bar graph analysis comparing protrusion length between control (HepG2 spheroid in MG), HepG2 and MRC-5-mixed spheroid, and HepG2 with high-density MRC-5 in the MG. Control (*n* = 3) compared with HepG2 with MRC-5 in MG (*n* = 3), *P* = 0.00053, and with MRC-5-mixed spheroid (*n* = 3), *P* = 0.00085. MRC-5-mixed spheroid (*n* = 3) compared with HepG2 with MRC-5 in MG (*n* = 3), *P* = 0.0014. Plotted is mean ± SD. Significance defined as *P* ≤ 0.05. **(C)** Schematic for modeling the LD with co-spheroid culture. HepG2 spheroids and MRC-5 spheroids are cultured in 384-well non-adherent plates for days and are either transferred together into a single well in a 96-well or 384-well plate and embedded in 50 μl MG for several days of culture. **(D)** Phase-contrast images of co-spheroid culture in a 96-well plate. Top left: Interface of liver spheroid (L) and MRC-5 spheroid (MRC-5) on day 6. Arrow shows interacting structures. Bar = 200 μm. Top right: high-magnification view on day 6. Arrow shows interacting structures. Bar = 100 μm. Bottom, day 7 cultures. Arrows show migrating strands and interacting structures. Bar = 100 μm. **(E)** Bar graph on days 5 and 6 demonstrating protrusion length and protrusion thickness. Plotted is mean ± SD. Significance defined as *P* ≤ 0.05. **(F)** Phase-contrast images of co-spheroid culture in a 384-well plate for days 3–9. “L” is the liver (HepG2) spheroid. Arrows demonstrate migrating hepatic cords between spheroids. Bar = 200 μm. **(G)** Same study as in **(F)** except days 9–13 phase-contrast and fluorescent images are shown. Left: high magnification of phase-contrast (top) and corresponding fluorescent (HepG2-GFP) images of green anchoring strands (arrows). Arrow demonstrates migrating strands. Right: phase-contrast images of day 11 (top) and day 13 (bottom) are shown. Arrows demonstrate hepatic cords anchored to MRC-5 spheroid. Bar = 100 μm. **(H)** Bar graph analysis of spheroid co-culture. Left: protrusion length on days 7, 9, and 11. Comparison of days 7 and 9 (*P* = 0.00063, *n* = 3) and days 7 and 13 (*P* = 0.00011, *n* = 3) is shown. Middle: protrusion thickness on days 7, 9, and 13. Comparison of days 7 and 9 (*P* = 0.0040, *n* = 3) and days 7 and 13 (*P* = 0.0019, *n* = 3) is shown. Right: comparison of protrusion thickness across all the culture systems developed on day 6. Spheroid 384-well co-culture (day 6, *n* = 3) compared with HepG2 with MRC-5 in MG (day 6, *P* = 0.0036, *n* = 3), with M-CM (day 6, *P* = 0.00007, *n* = 3), with MRC-5-mixed spheroid (day 6, *n* = 3, *P* = 0.007, *n* = 3), and with spheroid 96-well co-culture (day 6, *P* = 0.00047, *n* = 3). We also compared spheroid 96-well co-culture (day 6, *n* = 3) with HepG2 with MRC-5 in MG (day 6, *P* = 0.00046, *n* = 3), with M-CM (day 6, *P* = 0.006, *n* = 3), and with MRC-5-mixed spheroid (day 6, *P* = 0.02, *n* = 3). Plotted is mean ± SD. Significance defined as *P* ≤ 0.05. **(I)** Phase-contrast image of co-spheroid liver and MRC-5 spheroid on days 3, 5, 7, 9, and 13. Days 3–5: spheroids interact, day 7-spheroid fusion and migration at spheroid edge, day 9—arrows demonstrate protrusions, day 13-arrows demonstrate protrusions, Bar = 200 μm. **(J)** Phase-contrast and fluorescent images of a fused HepG2 and MRC-5 spheroid. Left; phase-contrast (day 13) image with arrows demonstrating protrusions. Double fluorescent (green: HepG2-GFP; red: MRC-5; arrows showing HepG2 (green) protrusions), red only (MRC-5), and green only (L, liver) with arrow showing HepG2 (green). Bar = 200 μm. ^∗^ is used to denote significance of experimental data.

To understand the process more fundamentally, we engineered several different systems that exhibit distinctive characteristics and morphogenetic features of liver 3D CCM. These systems include the following: (1) spheroid surrounded by matrix with stromal cells (Figure 1), (2) mixed spheroids (liver and fibroblasts) (Figure 2), (3) spheroid Matrigel (MG) droplet culture with fibroblast-conditioned medium (Figure 3), and (4) co-spheroid culture with a liver spheroid and a fibroblast spheroid in MG (Figure 5). These systems are distinguishable based upon the length, thickness, and kinetics of 3D CCM. This collective migration process encompasses several “modes” of 3D CCM, which we have modeled within the manuscript. These modes include the following: (1) co-migration in which two or more distinct cell types migrate together, (2) branching morphogenesis in which cells collectively migrate and undergo serial and patterned branching, (3) interstitial migration in which the epithelial cell type collectively migrates between tissues in 3D, and (4) thick strands or cords invading mesenchyme, which are distinct from thin, single cell strands. When testing different mesenchymal cell types, we more thoroughly define the role for MRC-5 (lung fetal) fibroblasts in inducing liver 3D CCM. In this model, we identify that TGFβ1 pathway plays a mechanistic role. To further model interstitial aspects of 3D CCM, we develop a novel spheroid co-culture system which demonstrates multiple aspects of CCM including thin and thick strand formation, interstitial migration, and spheroid fusion. These systems enable robust modeling of liver 3D CCM which will improve our molecular and cellular understanding of liver organogenesis, cancer, and therapy.

## Materials and Methods

### Reagents/Materials

Dulbecco’s modified Eagles’ medium (DMEM) (Cat. #: 10569010), fetal bovine serum (FBS) (Cat. #: A3160701), penicillin-streptomycin (Pen-Strep) (10,000 U/ml) (Cat. # 15140122), and 0.05% trypsin-EDTA (Cat. #: 25300062) were purchased from Thermo Fisher (Waltham, MA, United States). Endothelial Growth Medium-2 (EGM-2) BulletKit^TM^ (Cat. #: CC-3162) and MSCGMT Human Mesenchymal Stem Cell Growth BulletKit (Cat. #: PT-3001) were purchased from Lonza (Basel, Switzerland). Growth factor-free [Matrigel (MG) Cat. #: 40230], collagen rat tail (Cat. #: 354236), and 384-well round-bottom ultra-low attachment spheroid microplates (Cat. #:3830) were purchased from Corning Incorporated (Corning, NY, United States). Luciferase assay system (Cat. #: E1500) was from Promega (Madison, WI, United States). Two-well culture inserts (Cat. #: 80209) were purchased from Ibidi (Martinsried, Germany). Aurum Total RNA Mini Kit (Cat. #: 7326820), DNaseI (Cat. #: 7326828), iTaq^TM^ Universal SYBR^®^ Green Supermix (Cat. #: 1725121), and iScript cDNA Synthesis Kit (Cat. #: 1708891) were purchased from Bio-Rad (Hercules, CA, United States). Tissue culture-treated 24-well plate (Cat. #: 702001), 75-cm^2^ polystyrene tissue culture-treated flasks (Cat. #:708003), 60 mm tissue culture-treated dishes (Cat. #: 705001), 96-well cell culture plate (Cat. #: 701001), six-well cell culture plate (Cat. #: 703001), 96-well PCR plates (Cat. #: L223080), and PCR plate covers (Cat. #: HOTS-100) were purchased from Laboratory Products Sales (LPS) (Rochester, NY, United States). Vybrant DiD Cell-Labeling Solution (Cat. #: V22887), Vybrant DiO Cell-Labeling Solution (Cat. #: V22886), and Vybrant Dil Cell-Labeling Solution (Cat. #: V22885) were purchased from Thermo Fisher. Thrombin derived from human plasma (Cat. #: T6884-100UN) and fibronogen derived from human plasma (Cat. #: F4883-500MG) were purchased from Sigma Aldrich (Missouri, MO, United States). All PCR primers were purchased from either Integrated DNA technologies (IDT), Sigma Aldrich, or Thermo Fisher.

### Cell Lines

HepG2 liver carcinoma cells (Cat. #: HB-8065), MRC-5 lung fibroblasts cells (Cat. #: CCL-171), and human embryonic kidney cells (HEK-293) (Cat. #: CRL-3216) were purchased from ATCC (Manassas, VA, United States). Bone marrow mesenchymal stem cells (BM-MSC) (Cat. #: PT-2501) and human umbilical vein endothelial cells (HUVEC) (Cat. #CC-2935) were purchased from Lonza^®^. Human foreskin fibroblasts (HFF) cells were a kind gift from Professor Stelios Andreadis (University at Buffalo).

### Antibodies

Mouse anti-human Albumin monoclonal antibody (sc-271604), mouse anti-human FOXA2 monoclonal antibody (sc-374376), and mouse anti-human AFP monoclonal antibody (sc-130302) were purchased from Santa Cruz Biotechnology (Dallas, TX, United States). Ki67 recombinant rabbit monoclonal antibody (SP6) (Cat. #: MA5-14520), goat anti-mouse IgG (H + L) cross-adsorbed secondary antibody, Alexa Fluor 488 (Cat. #: A-11001), and goat anti-rabbit IgG (H + L) cross-adsorbed secondary antibody, Alexa Fluor 594 (Cat. #: A-11012) were purchased from Thermo Fisher.

### Engineering a Stable GFP/Firefly Luciferase HepG2 Cell Line

HepG2 cells (p10) cultivated at 200,000 cells/well in a six-well plate, were transfected with a third generation lentivirus bearing a transgene [ubiquitin-fluc2-egfp (Human Ubiquitin promoter, Firefly luciferase 2, enhanced green fluorescent protein)], as done previously (Parashurama et al., 2016) at an MOI of 5. To select for stably expressing cells, HepG2-Fluc2-eGFP cells were expanded, sorted by fluorescence activated cell sorting for high expressing cells, and replated, and then serial sorted two more times over a 4-week period, expanded, frozen in aliquots, and used in all experiments.

### Barrier Migration Assay

Briefly, cells were seeded into a two-chamber Ibidi co-culture system. This system consisted of two 80 μm/cm^2^ regions, favorable for cell adhesion, separated by a 500 μm gap. Eighty thousand cells were seeded into each region overnight. The following day the barrier was removed for induction of migration; ×10 images were captured every 30 min over a 24-h time period using phase-contrast microscopy. For experiments examining heterotypic cell interactions, control experiments consisted of the same cell type seeded adjacent to itself. Experimental conditions were composed of two different cell types seeded adjacent to each other. For conditioned media experiments, the control experiment consisted of the same cell type seeded adjacent to itself in serum containing DMEM media. The experimental condition consisted of the same cell type seeded adjacent to itself in conditioned media.

### Transwell Cell Migration

Cell migration was determined using 8 μm pore size transwells (Corning Incorporated) for 24-well plates. One hundred thousand HepG2 cells expressing GFP/Fluc were seeded onto the upper insert of the Transwell chamber. MRC-5 fibroblasts were added at varying concentrations below as a chemottractant. After 24h at 37°C, non-invading cells were removed using a wet cotton swab. Migrating cells on the lower surface were imaged using fluorescence microscopy (×10) and were subsequently analyzed and quantified. Images were obtained at ×10 magnification.

### Preparation of Conditioned Medium From Cell Lines

Adherent cell lines were seeded into a T-175 tissue culture-treated flask at a seeding density of 5,000 cells/cm^2^, incubated for 72 h in cDMEM. The total volume of media in culture was 15 ml. Cell culture medium is collected in a 15-ml tube after 72 h, centrifuged at 1,200 rpm for 5 min (debris removal), and filtered through a 0.2-μm filter to ensure sterility. The fresh medium is then added to different culture systems.

### Preparation of Agarose-Coated Microwells

Sterile, 1 wt.% agarose (1 g/100 ml distilled H_2_0) is prepared, warmed to liquid phase, and pipetted (50 μl) into each well of a 96-well plate (Corning Incorporated). The plate is allowed to cool (25°C for 20 min) prior to cell seeding.

### Spheroid Formation Assay

HepG2 and HEK 293T cells were cultivated in a T-75 flask with complete growth medium (cDMEM). DMEM was supplemented with 10% FBS and 1% Pen-strep and incubated at 37°C and 5% CO_2_ with medium changed every other day with a PBS. Cells were harvested with trypsin at 80% confluence (5 ml of 0.05% trypsin-EDTA) solution for 5–10 min followed by addition of medium, centrifuged (300 × *g* for 5 min), washed with PBS, resuspended in cDMEM, counted with a hemocytometer, and diluted (10,000 cells/ml of growth medium). With intermittent mixing, 100 μl (1,000 cells) of cell mixture is added to each well in an agarose-coated 96-well plate. The plated is sealed with parafilm, followed by centrifugation (1,000 rpm for 5 min). The parafilm was removed and the plates were incubated in 37°C and 5% CO_2_ for 72 h and imaged after every 24h using phase-contrast and fluorescent microscopy.

### Fluorescent Dye-Labeling of Cell Lines

Cell lines were harvested (0.05% trypsin) and adjusted to a final concentration of 1 × 10^6^ cells/mL in serum free DMEM. Next, 5 μl of either Vybrant DiD (red), Dil (yellow), or DiO (green) cell-labeling solution, respectively, is added to 1 ml of cell suspension in a microcentrifuge tube, incubated before for 20 min at 37°C, and centrifuged at 1,500 rpm for 5 min. The remaining cell pellet is then washed twice in fresh cDMEM and used in various cell culture systems.

### Spheroid Cultivation in Extracellular Matrices (Collagen, MG, Fibrin)

HepG2 spheroids were cultivated in 96- or 384-well or agarose-coated plates. Care was taken to pipette the gel solution into each well to prevent injury to the HepG2 spheroids. For MG-spheroid culture, an equal volume of MG was introduced for a 1:1 dilution. Additionally, premixed MG/collagen blends (mixed on ice) or fibrin were also introduced into each well. For experimental models with cells within the mesenchyme, BM-MSCs, HFF, MRC-5 cells, HUVECS, and HEK cells were premixed with a matrix component before being added at a total of 20,000 cells/well to each well unless otherwise described. Extracellular matrix-bearing plates were incubated for 30 min to ensure proper gel solidification followed by cDMEM addition.

### Collagen and Fibrin Gel Formation

Collagen gel (CG) (2 mg/ml) was prepared as suggested by the manufacturer. To make CG gels, rat tail CG was diluted with precalculated amounts of deionized water, 10 × PBS, and 1 N NaOH on ice, for a final concentration of 2 mg/ml. Fibrin hydrogels were made by premixing fibrinogen (3.25 mg/ml) and thrombin (12.5 Units/ml) in a 4:1 ratio on ice. The final solution contains equal amount of fibrinogen and thrombin, thus forming fibrin. Fibrin gels polymerize quickly at room temperature for spheroid-embedding applications.

### Spheroid Collection

Spheroids were collected from 96-well or 384-well plate culture, transferred to a 15-ml tube for spheroid settling, rinsed in cDMEM, and seeded as described below.

### Spheroid MG Outgrowth Assay

A MG-coated six-well plate was made by seeding 800 μl/well of diluted (1:14 diluted) MG onto six-well plates, followed by incubation for 30 min. HepG2 spheroids were collected (described above) and seeded as 15 spheroids per well in matrix-coated six-well plates. Spheroids attached to the plate overnight before purified conditioned medium was introduced into the plate the following day. Outgrowth-based migration was then observed over a 24-h period.

### Mixed Spheroid Formation

HepG2 and HFF cells are mixed together in a 1:1 ratio (20,000 cells total) to form compact spheroids in agarose-coated 96-well plates cultivated in cDMEM for 24h. These multi-cellular spheroids are then embedded in different matrices. In some cases, one or both cell populations are dye labeled, as described above.

### Spheroid MG Droplet Migration Assay

HepG2 and HEK spheroids were harvested (above), one spheroid at a time, in 15 μl MG, and seeded onto a 60-mm tissue culture-treated dish. MG droplets were solidified in the incubator for 30 min, and various medium types were added to the dish, with spheroids incubated for several days and analyzed as described.

### Spheroid Collagen Gel Droplet Migration Assay

The collagen droplet assay was performed similar to the MG droplet assay. Fifteen-microliter droplets of CG on ice, containing individual spheroids, were seeded onto 60 mm tissue culture-treated dishes. The droplets were incubated for 30 min and exposed to various culture medium conditions.

### Diluted Conditioned Medium Assay

M-CM (described above) was diluted by various ratios using cDMEM at ratios of 1 CM:1 cDMEM and 1 CM:7 cDMEM and were prepared and introduced to the matrix-embedded spheroids.

### MRC-5 Spheroid Formation Assay

MRC-5 cells were grown in a T-75 flask cDMEM medium, incubated at 37°C and 5% CO_2_ with medium changes performed every other day. Cells were passaged at 80% confluency and harvested with 5 ml of 0.05% trypsin-EDTA, washed, centrifuged (300 × *g* for 5 min), counted, and resuspended at 2 × 10^5^ cells/1 ml of cDMEM. With intermittent mixing, 50 μl of cell mixture was added to each well in an agarose-coated, 384-well ultra-low attachment plates. The plate was sealed with parafilm, followed by centrifugation (300 × *g* for 5 min). The parafilm was removed and the plates were incubated in 37°C and 5% CO_2_ for 72 h and analyzed as described.

### Spheroid Co-culture in MG Assay (384-Well Plate)

HepG2 spheroids were harvested (described above). Ten microliters of medium containing a spheroid were then added to the preexisting MRC-5 spheroids in a 384-well plate, one spheroid at a time, ensuring that each well contains both kinds of spheroids. Forty microliters of MG was added to each well, resulting in a 1:5 dilution of MG in co-culture.

### Histology

Spheroids were harvested from the 96- and 384-well plates, fixed in 4% paraformaldehyde for 30 min at room temperature, and embedded in agarose (2 wt.%) prior to paraffin embedding. Paraffin-embedded blocks were then sectioned at 10 μm. Antigen retrieval was performed by heating rehydrated section in 1 × Tris-EDTA buffer solutions for 20 min in microwave. Slides were then used for subsequent staining. Paraffin-embedded 10-μm sections were also stained with eosin and hematoxylin [Eosin Y Cat. #: (DcE-40), hematoxylin Cat. #: (DcH-48)] and mounted with medium before microscopy.

### Immunostaining of Monolayer and 3D Spheroid Culture

For detection of intracellular localization of albumin, AFP, FOXA2, or Ki67, cells cultured on tissue culture-treated dishes were washed once with PBS and then fixed with 4% paraformaldehyde, permeabilized with 0.1% Triton X-100 in PBS, and blocked with 1% BSA in PBS for 30 min. Plates were incubated with primary antibodies overnight, rinsed before incubation with secondary antibody (Alexa Flour 488, Thermo Fisher) for 1 h, incubated with 4,6-diamidino-2-phenylindole (DAPI) for nuclear detection for 10 min at room temperature. An additional staining protocol was developed and optimized to stain the whole mount of HepG2 spheroids in suspension. Briefly, HepG2 organoids were fixed in 4% PFA for 1 h and then blocked for 2 h in 1% BSA. The spheroids were then incubated with primary antibody at 5 μg/ml with gentle agitation at 4°C overnight. The following day, spheroids are washed three times with 1% PBST (each wash at 20 min) under gentle agitation at room temperature. Secondary antibody (Alexa Flour 488, Thermo Fisher) was then added for incubation at 4°C overnight and washed out as described above. DAPI incubation (10 min) was used to counterstain before the images were obtained. For immunolabeling of spheroids that generated protrusions in the presence of MRC-5 CM, the same protocol for organoid staining was used. Ki67 monoclonal primary antibody was used to stain the migrating spheroids overnight at 4°C. The following day, secondary antibody (Alexa Flour 594, Thermo Fisher) was applied again for an additional incubation overnight at 4°C before DAPI incubation (10 min) and subsequent imaging.

### Phase and Brightfield Microscopy

For cellular imaging, spheroids in various formats were imaged during morphogenesis using both phase-contrast and fluorescence microscopy. For phase microscopy, cell lines and spheroid cultures were imaged with a benchtop microscope (EVOS Fluorescent, phase-contrast microscope, #AMEFC4300R, 1,360 × 1,024 pixel density) at ×4, ×10, and ×20 or using a Zeiss Axio fluorescence microscope (SE64, 1,344 × 1,024 pixel density) equipped with Axiovision Software (v4) and analyzed using Image J^1^. Specifically, for live imaging of outward liver cell spheroid migration inside a 384-well ULA spheroid plate, images were taken at × 4 of individual wells under normal environmental conditions (25°C, 20% O_2_). In addition, live spheroid imaging inside MG droplets was also carried out at 25°C (20% O_2_). Images were obtained at the focal point of migration along the edge of the spheroid. Spheroid imaging was performed precisely at the same time daily to ensure accurate readings accounting for changes in shape characteristics (area, perimeter, cord length, and other parameters of interest).

### Fluorescence Microscopy

Cells were visualized under fluorescence microscopy using a standard filter for green (488 nm), blue (450 nm), or red fluorescence (594 nm). Images were captured using a Zeiss Axio fluorescence microscope (SE64, 1,344 × 1,024 pixel density) and equipped with Axiovision Software (v4) and analyzed using Image J. Fluorescence was obtained from LED light cubes specific to certain wavelength excitation and emission. Exposure time was manually set based upon signal-to-background ratio and was maintained constant within an experimental sample. The optimum algorithm was used to optimize grayscale values for minimum and maximum intensities. In mixed spheroid culture systems, to determine collective cell migration (yellow overlay), images were increased in brightness to overlay fibroblast (red) and liver cell lines (green). Images were obtained at the focal point of migration along the edge of the spheroid. Spheroid imaging was performed precisely at the same time daily to ensure accurate readings accounting for changes in shape characteristics (area, perimeter, cord length, etc.). Contrast changes made in separate channels were applied evenly across the entire image for that channel. In addition, color balance was made uniform across the entire images analyzed separately. Images obtained were.zip files, which could be formatted into.tiff files using Image J.

### Reverse Transcriptase Polymerase Chain Reaction

For each experimental condition, cell lysates were collected using the Aurum Total RNA Mini Kit. Total RNA was isolated from duplicate or triplicate samples, and concentrations were measured using the NanoDrop One/One Microvolume UV-Vis Spectrophotometer (Thermo Fisher). CDNA was synthesized using the iScript cDNA synthesis kit (Bio-Rad) and made using an Eppendorf 5331 MasterCycler Gradient Thermal Cycler (Eppendorf) with 5 ng of RNA for each planned qRT-PCR reaction. Each sample was plated in triplicate in a reaction volume consisting of 10 μl per well. Each well had 5 μl of iTaq^TM^ Universal SYBR^®^ Green Supermix, and forward and reverse primers at a concentration of 300 nM. The qRT-PCR reactions were run for 40 cycles in a Bio-Rad C1000 Touch Thermal Cycler. Gene expression analysis was conducted utilizing the delta-delta-Ct method, with GAPDH/B-actin used as normalization housekeeping gene(s). Primer sequences are reported in the Supplementary Information.

### Analysis of Spheroid Migration

Image J was used to determine relative properties of the migrating spheroids. Images were uploaded to Image J. A scale bar was set for each image uploaded before a subsequent analysis was performed. The length feature in Image J was used to determine protrusion length and thickness in the various experimental conditions. The count plugin feature was used to determine the number of cords or emerging strands in different fields of view and over time. The trace plugin feature in Image J was used to outline the edge of the spheroids over time to estimate the growth of the spheroid. The Skeletonize3D plugin in Image J was used to analyze the branching phenotype observed in migrating spheroids. Specifically, for analysis in skeletonize3D plugin, images were first converted to 8-bit grayscale, then a threshold was applied to isolate only the branching at the edge of the spheroid before skeletonize 3D was used to carry out branching analysis.

### Statistics

Data collected was reviewed and analyzed by GraphPad Prism (version 7) or Microsoft excel. Student’s *t*-test was used to determine statistical differences between two independent groups (*P*-value set at <0.05).

## Results

### Mesenchymal Stem Cells Induce Liver Cell Migration but Not in 3D Models of the Liver Diverticulum

We modeled the LD (Figures 1A,B) by modeling both the liver epithelium and the surrounding mesenchyme. The liver epithelium was modeled with a liver spheroid composed of HepG2, a hepatoblastoma cell line that expresses an immature gene expression profile, instead of hepatic endoderm/migrating hepatoblasts that are present in the LD. The surrounding mesenchyme was modeled with extracellular matrix hydrogels with stromal/mesenchymal cells. We began with hMSCs and HUVECs. hMSCs and HUVECs have been used to model the liver bud (E9.5) but not the LD (E8.5) (Takebe et al., 2017). To determine whether hMSC can induce cell migration *via* paracrine mechanisms, we employed a wound-healing model (Ibidi two-well culture insert). In control conditions (HepG2 alone), no changes were observed. When hMSC or HUVEC were added to the adjacent island, we observed significant changes in both border closure rate (HUVEC and MSC conditions) and liver-specific border area (MSC only) (Supplementary Figures 1A,B). Next, we performed a transwell assay, and liver cells (top) migrated toward the MSC (bottom), demonstrating a statistically significant induction of migration (Supplementary Figures 1C,D). To assess 3D migration, we employed a 3D spheroid liver diverticulum migration model (Figures 1C,D). 3D spheroids formed uniformly (Figure 1D), and cell dilution/labeling studies demonstrated that individual cells uniformly drive spheroid formation (Figure 1E). Whole spheroid immunostaining demonstrated homogenous albumin (Alb), alpha-fetoprotein (AFP), and Foxa2 expression (Figure 1F and Supplementary Figure 2), as expected. Gene expression analysis of 3D spheroids, compared with controls, demonstrates a decrease in Foxa2 transcriptional expression, but no significant changes in AFP, albumin, and TTR (Figure 1G), and histological analysis demonstrates no central necrosis by day 3 (Figure 1H). Using this 3D culture, we modeled the STM that is present in the LD with 20,000 hMSC (dye-labeled) within MG (Figure 1I). While we expected the liver cells to migrate toward the MSC, we instead observed that the hMSC migrated toward the liver spheroids within 24h and over a 3-day period (Figure 1J and Supplementary Figure 3A) and the data demonstrated no changes in circularity and decreasing distance from hMSC and liver spheroid (Figure 1K). To ensure that conditions under which MG did not lead to migration, we tested several conditions of gel formation including testing time and temperature of incubation, and the same effect was observed (data not shown). When we replaced hMSC with HUVEC (Jung et al., 2017; Pettinato et al., 2019), we observed HUVEC aggregation with no distinct liver cell migration (Supplementary Figures 3B,C). This data suggested that both MSC and HUVEC do not induce liver cell migration in 3D liver spheroids. We wanted to evaluate whether hMSC could be replaced by fibroblasts in our LD model. Employing *in vitro* migration models at first, we observed HFF not only migrated toward liver cells but also induced liver cell migration in 2D models (Supplementary Figure 4). Therefore, within our 3D LD model, we employed HFF within MG as an alternative to MSC or HUVEC (Figure 1L). Interestingly, we observed evidence of perpendicular cellular protrusions and cellular strands at the edges of the liver spheroid in the presence of the HFF by day 12 (Figures 1M,N). While we had to wait until day 12 to observe migration, we did not observe similar migration in the hMSC- or HUVEC-liver spheroid systems on day 12 (data not shown). While we suspected that HFF joined the spheroid and were part of the migrating strands, it could not be fully determined.

### MRC-Induced Fibroblasts Enhance 3D Liver Collective Cell Migration in a Mixed Spheroid Model

Since we observed migration when HFF mixed with HepG2 cells, we hypothesized that creating mixed spheroid co-culture (HFF and liver), that mimic the heterotypic interactions in the liver bud (E9.5-10), may enhance the kinetics of 3D CCM formation (Figure 2A). In mixed spheroids with HFF and HepG2, we observed rapid compaction improving to 24h compared with 3 days in liver spheroids (data not shown). To determine individual cell fate within the mixed spheroid, we performed double labeling of liver (eGFP) and HFF (DiI dye) cells after embedding in MG. Serial *in vitro* microscopy demonstrated uniform, migrating strands that increase with time (Figure 2B) compared with the negative control condition (no MG). To determine if co-migration of both cell types occurred, we performed single-well fluorescent imaging of labeled cells and adjusted images to maximum brightness to clearly visualize migrating strands at the edge. The data demonstrated a large number of migrating strands, including red strands, yellow strands (both green and red), and in a few cases, green strands (HepG2) (Figure 2C), demonstrating statistical differences between presence and absence of MG (Figure 2D). We analyzed effects of migration in different extracellular matrix conditions. We found that with culture of mixed spheroids in collagen gels, fibroblasts appear to migrate in an elongated fashion (Supplementary Figure 5A, left). In fibrin gels, both fibroblasts and HepG2 cells individually take an elongated morphology (Supplementary Figures 5A (right), 6), while in collagen/MG mixtures, we observe fibroblast migration and statistically significant nodule formation (Supplementary Figures 5A(middle),B). Despite the varied morphogenesis we observed when varying matrix composition, there was no observed 3D CCM of liver cells nor was co-migration of two cell types observed.

We hypothesized that testing an alternate fibroblast type could increase 3D migration in our liver spheroid model. MRC-5 fibroblasts are derived from human fetal lung tissue and unique among human fibroblast lines to induce epithelial cell scattering in 2D *in vitro* models (Stoker and Perryman, 1985). Thus, we hypothesized that MRC-5 fibroblasts will induce enhanced liver 3D CCM when used in our LD model with mixed spheroids. We observed significantly more migration (length and number of protrusions) in the case of MRC-5 condition on day 3, compared with the HFF condition (compare Figure 2E with Figures 2B,C,F and Supplementary Figure 7). Since TGFβ pathway potently affects hepatic cord formation (Weinstein et al., 2001), we wanted to evaluate the effects of this pathway on 3D CCM. Therefore, we added TGFβ1 growth factor to mixed spheroids with MRC-5 cells, and we observed a statistically significant increase in the projected area of migration in the MRC-5, condition compared with the control (compare Figure 2G with Figures 2H,I). These experiments suggest that MRC-5 cells specifically enhance liver 3D CCM, with further enhancement by TGFβ1, in mixed liver spheroids.

### MRC-5-Conditioned Medium Induces 3D CCM Within the *in vitro* Liver Diverticulum Model

While mixed (liver and MRC5) spheroids did result in evidence of co-migration (both red and green), we wanted to determine how MRC-5 resulted in an enhanced 3D liver CCM. We evaluated M-CM, which has previously been shown to increase liver cancer cell migration (Ding et al., 2015). We first performed *in vitro* migration assays employing the Ibidi system as above and the addition of M-CM resulted in statistically significant HepG2 border migration (Supplementary Figures 8A,B). Next, we performed a transwell assay, which shows liver cell migration in response to M-CM. We found a statistically significant increase in migration of liver cells in response to MRC-5 cells compared with hMSC (Supplementary Figures 8C,D). To determine whether this effect was specific to the MRC-5 cells, we performed a classic outgrowth assay in which liver spheroids were cultured in control medium, HepG2-conditioned medium, HFF-conditioned medium, and M-CM. The data demonstrated clear, statistically significant increase in spheroid outgrowth in the presence of M-CM after 24h of outgrowth (Supplementary Figures 9A,B). The data also demonstrated a downregulation of E-cadherin at the edge of the spheroid (Supplementary Figure 10), consistent with a migrating phenotype. This outgrowth assay demonstrated migration on tissue plastic, which did not fully represent 3D migration. To test 3D migration, we developed a 3D migration assay by suspending liver spheroids in MG droplets (Figure 3A and Supplementary Figure 11) (Lancaster et al., 2013). In this system, we first determined how the strength of M-CM affects 3D cell migration. We found that from days 4 to 11, M-CM led to 3D CCM (Figure 3B) and resulted in greatly increased migration in a 2D outgrowth assay (Supplementary Figure 11). On day 7 in MG droplets, we observe submicron cellular strands including long strands with small branching, long strands without branching, thicker strands, and strands with multiple levels of branching (Figure 3B, left). On the other hand, by day 11 of culture, the migrating strands appeared to be interconnected, formed a sheet of cells, and attached to the dish, and the cells had spread with visible nuclei (Figure 3B, right). Even though these migrating strands appeared to touch the dish, the cells exhibited branching and elongated morphologies on day 11 that could be clearly distinguished from spheroid outgrowth assay [compare Figure 3B with Supplementary Figure 9A (M-CM condition)].

We then hypothesized that that M-CM may induce migration *via* TGFβ, based on our data in Figure 2. We tested the TGFβ pathway inhibitor A83-01, which inhibits TGFβ, Activin, and Nodal signaling pathway, by inhibiting ALK4, ALK5, and ALK7 receptors and prevents SMAD2/3 phosphorylation. We observed a statistically significant, and dose-sensitive, inhibition of migration (Figures 3C,D). To further elucidate how M-CM functions, we performed dilution studies (1:1, 1:7, M-CM first day only) and demonstrated statistically significant differences in 3D CCM as a function of dilution (Figures 3E,F). Although M-CM at a ratio of 1:7 surprisingly still resulted in 3D CCM, when we diluted M-CM at a ratio of 1:19, we observed generalized outgrowth but no evidence of branching migration (Supplementary Figure 12). During 3D CCM, nuclear imaging distinguishes cell protrusion from cell migration. Cell nuclei were not visible during MCM-induced migration, but when we performed nuclear (DAPI) and Ki67 staining on day 7 of spheroid culture, we found nuclear staining within 3D protrusions, and heterogeneous areas of Ki67 staining suggesting proliferation may be involved in some of the protrusive effects (Supplementary Figure 13). We tested conditioned medium from other cell types and observed no effects but observed that MRC-5 also induces 3D CCM in HEK cells by day 11, which serves as a positive control (Supplementary Figure 14). To evaluate the effect of matrix (and potentially stiffness) on 3D migration, we tested the same liver spheroid droplet model in a CG gel. Interestingly, by day 7 of culture, we observed highly linear, micron- or submicron-thick, cellular strands extending from the liver spheroid in several experimental replicates [compare Figure 3B (Day 7) with Figure 3J (Day 7)]. We clearly observed linear strands with less branching than observed in MG (Figure 3J and Supplementary Figure 15). We demonstrated a statistically significant increase in protrusion length and cord count with M-CM in CG compared with the control medium (Figure 3K). When we compared the MG and CG conditions, we found that protrusion thickness was significantly reduced in the CG condition, the number of protrusions was comparable in both the CG and MG conditions, and the CG condition had statistically less branching than the MG condition (Figure 3L). Collectively, these data demonstrate robust collective cell migration within the liver diverticulum model of MRC-5-conditioned medium, controlled by M-CM dilution and matrix conditions.

### Effects of 3D Migration on Gene Expression

We performed gene expression analysis of liver spheroids in control and M-CM conditions on day 7 normalized to day 3 (liver spheroid). We analyzed genes associated with differentiation and migration. In the presence of M-CM, on day 7, we observed no significant changes in albumin and AFP expression (Figure 4A), while for key transcription factors, we observed a trending increase in Foxa2, a trending decrease in HNF4a (global transcriptional activator), and a significant decrease in CEBPa (metabolic functions) (Figure 4B). In terms of EMT transcription factors, we observed no change in SNAIL2 expression and a significant upregulation of TWIST1 (Figure 4C). We also observed a trending downregulation of *E*-cadherin and *N*-cadherin expression (Figure 4D).

### Engineering Fibroblast Density and an Optimized Cultivation System for Increased 3D Collective Cell Migration

Although we consistently observe liver 3D CCM with M-CM, we wished to improve *in vitro* modeling by addressing how cellularity within the STM could affect cell migration and induce 3D liver CCM. We first used our earlier LD model (Figure 1I) and increased the MRC-5 cellular density by 50%. At these higher densities, we found that small islands of MRC-5 fibroblasts formed within the MG, and in response, thick liver spheroid-derived cords protruded from the spheroid to these fibroblasts islands to form thick strands containing both cell types (Figure 5A, days 9–12). These cords formed by day 9, thickened to an average of 115 μm by day 12 (Figure 5B, protrusion thickness), and demonstrated significantly longer strands then the mixed spheroids and control (no MRC-5). We also observed that the liver spheroid exhibited radial migrating strands away from these thick strands at the edge of spheroids (Figure 5A, day 12), but new thick strands did not form after day 9. To further increase cellular density of the MRC-5 fibroblasts and obtain a system in which migration occurred faster, we changed the experimental system to a co-spheroid culture in which liver and MRC-5 spheroids are cultured together in MG (Figure 5C). This co-spheroid also better modeled the cell dense STM that occurs in the LD (Figures 1A,B). Surprisingly, we found that when spheroids were placed within 150 μm from each other in a 96-well plate, we observed a trending increase in filopodia extension with concomitant cell migration by day 6 (Figures 5D,E). To improve spheroid interactions, we changed the cultivation system from a 96-well to a 384-well format. In this cultivation system, the liver spheroid is transferred from the well and there is a minor loss of circularity (Figure 5F, day 3, “L” spheroid). Here, we observed multiple thin strands/cords that form on day 9 (Figure 5F, day 9). They originate from the liver spheroid and then they move toward the spheroid and anchor to the MRC-5 spheroid (Figure 5G). This is highly reminiscent of the 3D finger-like projections of hepatic cords that form during liver organ development (compare Figures 5F,G with Figure 1A), based upon the reported dimensions of width and length in published liver bud studies (Matsumoto et al., 2001; Suzuki et al., 2006; Watt et al., 2007; Ludtke et al., 2009). We observed a significant increase in protrusion length (Figure 5H, left) and protrusion thickness over time (Figure 5H, middle). We observed statistically significant differences between the co-spheroid culture systems, and all other systems developed, with the co-spheroid culture demonstrating the thinnest strands (Figure 5H, right). In this co-spheroid culture system with a 384-well plate instead of a 96-well plate, we observed novel findings. From days 3 to 7, we observed fusion of the two spheroids (Figure 5I), followed by formation of 3D CCM of radial cell strands from days 7 to 13 (Figure 5I). These strands were maximal at day 9 but retracted by day 13. We performed fluorescent labeling and imaging of spheroids to understand spheroid interactions between liver spheroid (green) and MRC-5 spheroid (red). Interestingly, we found that the liver spheroid engulfs the MRC spheroid (Figure 5J), from days 3 to 7, using fluorescent imaging, which requires liver cells migrating interstitially over MRC-5 spheroid cells. This engulfing process occurs without an overt change in size, suggesting that cellular packing density is increased. Two-color imaging enables us to identify that the cellular projections from the spheroid are primarily of hepatic origin (Figure 5J). Minimal red fluorescent strands are seen, indicating that nearly all radial migrating strands are from the liver spheroid. Taken together, our data demonstrate that modeling the STM at high density results in significant morphogenetic events, such as thick strand/cord formation, thin strand/cord formation, interstitial migration, and spheroid fusion.

## Discussion

Liver 3D CCM is critical in several scenarios, including the following: (1) hepatic cord formation during hepatic endoderm migration, (2) early fetal hepatocyte interstitial migration during liver expansion, (3) hepatocyte migration during liver repopulation, (4) spread and metastasis in hepatocellular carcinoma, and (5) potentially other pathologic processes, like nodule formation or bridging fibrosis. We hypothesized that modeling the LD can lead to improved 3D CCM modeling. Using a liver cancer cell line that displays an immature phenotype, we alter both spheroid composition and mesenchyme composition to reproducibly recreate several modes of liver 3D CCM. These systems we have devised and analyzed include the following: (1) spheroid surrounded by matrix with stromal cells (Figure 1), (2) mixed spheroids (liver and fibroblasts) (Figure 2), (3) spheroid MG droplet culture with fibroblast-conditioned medium (Figure 4), and (4) co-spheroid culture with a liver spheroid and a MRC-5 fibroblast spheroid in MG (Figure 5). By recreating distinct aspects of 3D CCM and liver morphogenesis, including co-migration, linear motion, branching morphogenesis, strand (cord) formation, and interstitial migration, we have expanded the repertoire of systems that further our understanding of 3D CCM. Importantly, our systems exhibit distinctive characteristics and morphogenetic features of liver 3D CCM, in terms of the length, thickness, and kinetics of 3D CCM, and we find that TGFβ plays a role in early 3D CCM and branching morphogenesis. These studies will further our molecular and cellular understanding of liver cell migration and improve treatments for HCC metastasis and liver cell therapy.

A key finding is that M-CM initiates 3D liver migration in both MG and CG systems. The MG studies demonstrate a model for branching morphogenesis, while the CG studies demonstrate linear and elongated protrusive growth. Our data also demonstrates that DAPI (nuclear) staining occurs in these protrusions, providing evidence for increased cell motility *via* collective migration, and that Ki67 staining (proliferation) is associated with some of the protrusions. Although 2D systems differ from 3D systems, previous HCC studies of 2D migration agree with our results (Ding et al., 2015). These previous studies of migration can be classified as non-classical EMT and are tissue and cell line specific. For example, in pancreatic cancer cell lines, M-CM has been paradoxically shown to inhibit cell migration, invasion, promote a more immunosuppressive phenotype, and coordinately increase cell polarity markers (Ding et al., 2018). In these cell migration studies, gene expression does not necessarily correlate with protein expression, suggesting underlying complexity and protein redistribution as a mechanism. Previous studies that analyze MRC-5 secretions led to the isolation of HGF (Stoker and Perryman, 1985; Schor et al., 1988), suggesting that HGF is a causative factor in migration with unclear mechanisms. HGF has been shown to promote migration of murine oval cells or hepatic stem/progenitor cells (Suarez-Causado et al., 2015), as well as Huh-7 and HepG2 cells (Meng et al., 2015) using traditional *in vitro* assays. Since TGFβ pathway is implicated in hepatic cord formation (Rossi et al., 2001) and HCC migration (Fransvea et al., 2008) and has shown to be involved in cross-talk with HGF pathway (Rossi et al., 2001), we evaluated TGFβ pathway inhibitors. We indeed identified that large effects in migration can be attributed to TGFβ-mediated 3D CCM (Figure 2H). The addition of A83-01, a broad inhibitor which inhibits TGFβ, Activin, and Nodal signaling pathway, by inhibiting ALK4, ALK5, and ALK7 receptors and preventing SMAD2/3 phosphorylation, resulted in a dramatic decrease in migration in a dose-dependent fashion (Figure 3C). Furthermore, analysis employing system biology techniques of the secretome and exosome of M-CM may find the mechanism by which M-CM functions. Since MRC-5 lung fibroblast cells were isolated at 14 weeks (∼E18 in mouse), fetal-like mesenchyme may be critical for 3D liver CCM. Thus far, modeling of STM in liver bud models has been accomplished with hMSC and HUVEC (Takebe et al., 2017), but many factors like matrix composition, matrix stiffness, and mesenchymal cell state still need to be explored. Studies continue to elucidate key molecules underlying the STM composition (Zhao and Duncan, 2005) which suggests that *in vitro* LD models can be improved in several ways (Coll et al., 2018).

The concept of co-migration is critical because co-migration of hepatic endoderm/hepatoblasts, STM cells, and endothelial cells occurs in the liver bud, after the liver diverticulum stage (Cascio and Zaret, 1991). Important commonalities between HepG2 and hepatic endoderm/early hepatoblasts is that both of these cells express liver-specific transcription factors, alpha-fetoprotein (AFP) and albumin (ALB) proteins, and have migratory capacity. AFP is a marker for immature cells, suggesting that HepG2 cells are immature and often present in tumors. While we understand that the cells are not identical, we do think that these cells have phenotypes in common. The factors that promote liver co-migration of liver and mesenchymal cells are poorly understood. Here, we determined the conditions under which co-migration occurs using a mixed spheroid system containing liver hepatoma cells with either HFF or MRC-5. Mixed liver spheroids have been previously used to improve viability of spheroids, and enhanced cancer invasiveness, as demonstrated by changes in gene expression and enhanced drug resistance (Jung et al., 2017). Furthermore, mixed systems have been shown to model the liver bud, which is the stage immediately after the LD stage (Takebe et al., 2013; Figure 1A). The mixed spheroids with HFF in MG resulted in co-migration (Figure 1M). When we employed stiffer matrix, like CG and fibrin gels, we observed extremely long, thin protrusions that were submicron and hair like. We used cell labeling to determine that these long thin protrusions do not demonstrate co-migration (Figure 2B and Supplementary Figures 5,6). This suggests that co-migration requires either soft hydrogels or specific components within the MG. Furthermore, in our co-migration models, we do not observe branching morphogenesis, as we do with M-CM studies, suggesting that supporting cells in mixed spheroids may suppress branching morphogenesis. It remains to be seen if co-migration together with branching morphogenesis can occur simultaneously. Our data also suggests that TGFβ1 increases the number of protrusions and the overall area of growth due to co-migration, but not branching. The factors that influence liver/mesenchymal co-migration are not known. However, in studies of squamous cell carcinoma, it was shown that Rho-ROCK-activated fibroblasts physically lead collective cell migration by secreting proteases (MMP) and generating tracks within the collagen matrix in which groups of cancer cells can follow (Gaggioli et al., 2007). We feel by improved modeling of various aspects of morphogenesis, we will be able to better tease out the multiple biomechanical and protein translational changes that result in morphogenesis and/or migration (Okuda et al., 2018) and use this to improve liver cell therapy and cancer therapies.

In our co-spheroid culture, we had several important findings. In the absence of spheroid fusion, we observed thin strands originating in the liver spheroid and interacting with the MRC-5 spheroid, very similar to what occurs within the developing LD. These cell strands or protrusions do not contain branches and anchor to the MRC-5 spheroid indicating morphogenetic processes and interstitial migration. We also observe an interesting case of mixed spheroids when the liver spheroid fuses with the MRC-5 spheroid (Figures 5I,J). In some cases, these self-assembled spheroids undergo fusion, which we term self-driven morphogenesis (Sasai, 2013). This is highly reminiscent of fused organoids that have received increased attention (Bagley et al., 2017; Xiang et al., 2017, 2019; Mansour et al., 2018) for stem cell biology and bioprinting (engineering) applications. In a recent study with spheroid fusion to model the developing gut demonstrate that the expected migration of hepatic endoderm progenitors did not occur (Koike et al., 2019), demonstrating the importance of LD models that induce liver CCM. Here, we demonstrate clear evidence of hepatic cells engulfing mesenchymal (MRC-5) spheroids, which likely requires interstitial, 3D CCM of liver cells migrating on top of MRC-5 fibroblasts. A physiological example in which an epithelial population engulfs a mesenchymal population, is during the process of hair follicle development (Millar, 2002). Similarly, spheroids of retinal progenitor cells (epithelial cells) can envelope spheroids of limbal MSC in culture (Kosheleva et al., 2020), consistent with our data of MRC-5 fibroblasts remaining inside during fusion. In terms of the mechanism of spheroid fusion, it was previously found that that tissue spheroids that were exposed to TGFβ-favored fusion with non-exposed spheroids, which was consistent with our data that MRC-5 cells may secrete TGFβ molecules (Hajdu et al., 2010). However, the roles for extracellular MG, signaling pathways, and cell and tissue mechanics still need to be determined. We speculate that these models can be used to investigate two morphogenetic structures that form in liver disease, including the following: (1) regenerative nodules in chronic liver disease and (2) bridging fibrosis in advanced stage fibrosis and cirrhosis. The fused spheroid system we have developed results in liver cell migration, but not co-migration. These are duct-like protrusions that are uniquely liver only, that do not branch, and whether the mechanism is chemical (TGFβ) or biomechanical (stiff mesenchymal core) is unclear.

There were several limitations to our study. Our cell type here was a common hepatoma cell line, but in the future, we will employ a cell that is more like the human-pluripotent stem cell-derived hepatic endoderm to enhance the models. We observed migration followed by branching morphogenesis in the presence of M-CM. A major question is whether nuclei also migrate in the structures. Our data collected of DAPI staining strongly suggests that at least some of these structures have nuclei (Supplementary Figure 10). Further analysis of nuclear staining at high resolution is needed to confirm the nature of these branching structures. From a methods point of view, it was challenging to recover spheroids from miniature plates like 96- and 384-well plates. This resulted at times in a slight loss of sphericity, and we attempted several approaches to transfer spheroids from one system to another, none of which were perfect. Our studies of extracellular matrix effects with M-CM tested a few types of matrix, but future studies will analyze the effects of extracellular matrix more vigorously, including studies of fibrillar vs. cross-linked collagen and independent studies of composition and stiffness. Finally, our MG droplet system lasts about 2 weeks, but then the matrix degrades significantly, and thus improved methods are needed for long-term MG droplet culture. Finally, as our systems were cultured in tissue culture plastic-based plastic/wells, it was challenging to perform high magnification of analysis. Future studies will be performed on glass for improved imaging, or with tissue clearing protocols followed by light-sheet or multiphoton microscopy. Nonetheless, we present several new systems which result in various forms of 3D CCM. We not only observe varying thickness and length of protrusions that can be further dissected mechanistically but we also demonstrate co-migration, branching morphogenesis, and interstitial migration, all of which are valuable for scientists modeling liver development, liver cell therapy, or liver diseases.

## Data Availability Statement

The original contributions presented in the study are included in the article/Supplementary Material, further inquiries can be directed to the corresponding author/s.

## Author Contributions

OO and OY obtained and analyzed the data, and wrote and approved the manuscript. CO, AK, WL, CS, and SR obtained and analyzed the data, and approved the manuscript. NP conceptualized, acquired the funding, investigated, supervised, wrote, edited, and approved the manuscript. All authors contributed to the article and approved the submitted version.

## Conflict of Interest

The authors declare that the research was conducted in the absence of any commercial or financial relationships that could be construed as a potential conflict of interest.

## Footnotes

1 https://imagej.nih.gov/ij/

## Supplementary Material

Supplementary Figure 1 **(A)** Phase-contrast images of 2D barrier migration assays between HepG2 and mesenchymal stem cells (HUVECS and MSCs) on at *T* = 0 h (top) and *T* = 24h (bottom). Time lapse performed every 30 min over 24h. **(B)** Left: bar graph analysis of migration demonstrates a significant increase in barrier closure in the presence of HUVECS (*P* = 0.00057, *n* = 3) and hMSCs (*P* = 0.00028, *n* = 3) as compared with HepG2 control (*n* = 3). Right: bar graph analysis of HepG2 border wall movement in the presence of MSCs (*P* = 0.047, *n* = 3) as compared with HepG2 control (*n* = 3). Plotted is mean ± SD. Significance defined as *P* ≤ 0.05. **(C)** Fluorescence image focused on a transwell during a transwell assay demonstrating HepG2-GFP migration alone (left) or in the presence of MSCs (right). Bar = 200 μm. **(D)** Bar graph plotting average number of HepG2-GFP migrating through transwell (*P* = 0.013, *n* = 3). Plotted is mean ± SD. Significance defined as *P* ≤ 0.05.

Supplementary Figure 2 HepG2 spheroids in suspension culture were collected and immunolabeled in suspension. Briefly, spheroids were harvested and fixed with 4% paraformaldehyde (PFA) at room temperature, permeabilized with 1% Triton X-100 and then incubated overnight with either alpha-fetoprotein (AFP) or FOXA2 monoclonal primary antibody. These markers are specific to liver lineage commitment. HepG2 spheroids were observed to positively express FOXA2 and AFP.

Supplementary Figure 3 **(A)** HepG2 cells are initially formed into spheroids using 384-well ULA plates over a 3-day culture period. Separately, MSC cells, premixed with MG are collected and seeded as 20,000 cells per microwell containing a HepG2 spheroid. Columns of images are replicates 1 and 2 with double fluorescent images on days 4 and 5. HepG2 (green), MSC (red). Bar = 500 μm. **(B)** HepG2 spheroids (L) were initially formed in suspension culture using 384-well round-bottom ultra-low attachment plates (1,000 cells per well). On day 3, spheroids were then embedded in MG (GFR) containing 20,000 HUVEC cells. Additional growth media to support HepG2 and endothelial cell proliferation, DMEM:EGM in a 1:1 ratio, was used to culture the embedded spheroids. **(C)** By day 6, no significant outward migration of HepG2 cells was observed into the ECM. Aggregates of endothelial cells were observed in the MG as clusters (black arrow) that showed no interaction with the HepG2 spheroid.

Supplementary Figure 4 A barrier migration assay to establish the effect of human foreskin fibroblasts (HFFs) on HepG2 collective cell migration. Briefly, an Ibidi^TM^ two-well insert was used to create a barrier between HFFs and HepG2 cells seeded 500 μm apart. After overnight incubation to allow for cell attachment, the insert was removed to allow for migration between the cell reservoirs. Collective migration of HepG2 cells is observed outwards in response to the presence of the HFFs present.

Supplementary Figure 5 **(A)** Phase-contrast images of HepG2/HFF-mixed spheroids. Left: in collagen gel (CG) on day 6, arrows show hair-like structures migrating. Middle: in CG/MG mixture, arrows show node-like structures in hydrogel. Right: in Fibrin hydrogel, hair-like structures are migrating. **(B)** Bar graph plotting node density within the mesenchyme, comparing HepG2/HFF spheroids grown in the CG and CG/MG condition (day 6, *P* = 0.0043, *n* = 3). Significance defined as *P* ≤ 0.05.

Supplementary Figure 6 HepG2 cells and human foreskin fibroblasts (HFFs) were initially separately dye-labeled green and red, respectively, and then mixed in a 1:1 ratio before being seeded into 384-well round-bottom ultra-low attachment plates. Cells formed a compact spheroid that was subsequently embedded in a premade fibrin hydrogel. Fibrin hydrogel was made by polymerizing fibrinogen (3.25 mg/ml) with thrombin (12.5 U/ml) in a 4:1 ratio. There was enhanced co-migration of HepG2 cells and HFF cells outwards into the fibrin matrix.

Supplementary Figure 7 Fluorescent images of days 2–4 of HepG2-GFP/MRC-5-mixed spheroids in MG. From left to right: HepG2 (green) cells and combined HepG2 (red) and MRC-5 (yellow) images. Replicates 1 (above) and 2 (below) are shown. Arrows show HepG2 and MRC-5 migration.

Supplementary Figure 8 MRC-5-conditioned media support liver cell migration in multiple 2D migration assays. **(A)** Phase-contrast images of 2D barrier migration assays between HepG2 cells (on both sides of the barrier) in the presence of MRC-5-conditioned medium (M-CM) at *T* = 24h in control (left) and M-CM (right) conditions. **(B)** Bar graph comparing residual area between control and M-CM conditions (*P* = 0.00085, *n* = 3). Plotted is mean ± SD. Significance defined as *P* ≤ 0.05. **(C)** Fluorescence image focused on a transwell during a transwell assay demonstrating HepG2-GFP migration alone, or in the presence of MRC-5 (and resulting M-CM), after 24h. **(D)** Bar graph analyzing transwell assay comparing control with MSC (*P* = 0.023, *n* = 3), MSC with MRC-5 MSC (*P* = 0.012, *n* = 3), and control with M-CM conditions (*P* = 0.0014, *n* = 3). Plotted is mean ± SD. Significance defined as *P* ≤ 0.05.

Supplementary Figure 9 Phase-contrast images of HepG2 spheroid outgrowth assay in control, HepG2 CM, HFF-CM, and M-CM (MRC-5 CM). **(A)** Spheroids cultured on MG (1:15)-coated plates. **(B)** Bar graph for spheroid outgrowth assay comparing area of spheroid growth (left) and growth perimeter (right) between control, HepG2-CM, HFF-CM, and M-CM conditions. Comparison of spheroid growth area for M-CM and control (*P* = 0.0023, *n* = 3), M-CM and HepG2-CM (*P* = 0.0030, *n* = 3), and M-CM and HFF-CM (*P* = 0.0029, *n* = 3) as shown. Comparison of growth perimeter between M-CM and HepG2-CM (*P* = 0.0030, *n* = 3) and M-CM and HFF-CM (*P* = 0.0029, *n* = 3) as shown. Plotted is mean ± SD. Significance defined as *P* ≤ 0.05.

Supplementary Figure 10 Spheroid outgrowth assay in control and M-CM medium with immunocytochemistry for *E*-cadherin. HepG2 spheroids seeded onto MG-coated plates (1:15 dilution) are allowed to attach (24h). For the top condition, the medium is changed to control medium (DMEM + 10% FBS) and to MRC-5-conditioned media (0.2 μm filtered as normal) for the bottom condition. Attached spheroids are incubated for an additional 24h, and then underwent immunocytochemistry for *E*-cadherin. Spheroids cultured in MCM showed irregular localization of *E*-cadherin around the migrating edge of the spheroid, suggestive of collective cell movement outwards. Spheroids cultured in the control condition, exhibited strong expression along the spheroid edge.

Supplementary Figure 11 Images obtained of day 7 HepG2 MG droplet culture systems in MRC-5-conditioned media (MCM) and control experimental conditions (serum containing DMEM). The MCM condition is a lighter pink color than the control condition which is supportive evidence for the degradation of the MG by the migratory HepG2 cells in response to the presence of the MCM.

Supplementary Figure 12 HepG2 spheroids cultured in 1:19 diluted MRC-5-conditioned media (M-CM) exhibit no matrix invasion. HepG2 spheroids formed in suspension culture using a 384-well round-bottom ultra-low attachment plate were harvested and seeded into MG droplets on tissue culture-treated 60 mm dishes. MG droplets were then cultured in 1:19 M-CM for an additional 4 days. On day 7, spheroids showed no significant outward migration into the extracellular matrix (ECM). The M-CM media typically supports extensive HepG2 migration.

Supplementary Figure 13 Ki67 staining of HepG2 spheroids in MG droplet culture. HepG2 spheroids formed in suspension culture using a 384-well round-bottom ultra-low attachment plate (3-day culture) were harvested and seeded into MG droplets on tissue culture-treated 60 mm dishes. MG droplets were then cultured in MRC-5-conditioned media (M-CM) for an additional 4 days. On day 7, droplets were fixed with 4% paraformaldehyde (PFA) at room temperature, permeabilized with 1% Triton X-100, and stained using monoclonal primary antibody, Ki67, a marker of proliferation. Spheroids treated with M-CM staining showed positive expression for Ki67 localized to migrating cords as compared with control, droplets cultured in serum-containing DMEM.

Supplementary Figure 14 Phase-contrast images on day 7 of HepG2 spheroid in MG culture treated with various conditioned medium conditions including M-CM (far left), control (DMEM, 10% FBS, 1% Pen-strep, left), HFF-CM (right), and HepG2-CM (far right). In addition, a separate cell line, human embryonic kidney cells, HEK, were made into spheroids, embedded into MG, and cultured in M-CM (days 7 and 11 are shown). Radial migration is apparent in HepG2 and HEK conditions where M-CM media are included.

Supplementary Figure 15 Collagen gel-embedded HepG2 spheroids on day 7 exhibit radial migration. HepG2 spheroids were formed in suspension using a 384-well round-bottom ultra-low attachment plate (3-day culture) were collected and seeded into collagen droplets on tissue culture-treated 60 mm dishes. Collagen droplets were then cultured in MRC-5-conditioned media (M-CM) for an additional 4 days. On day 7, migrating spheroids were observed and shown to exhibit thin radial protrusions into the matrix that was consistent among eight individual repeats.
