# Supplementary figures and images for: Modeling Liver Organogenesis by Recreating Three-Dimensional Collective Cell Migration: A Role for TGFβ Pathway
Source: Front Bioeng Biotechnol. 2021 Jun 15;9:621286. doi: 10.3389/fbioe.2021.621286 (PMC8239196; doi:10.3389/fbioe.2021.621286)

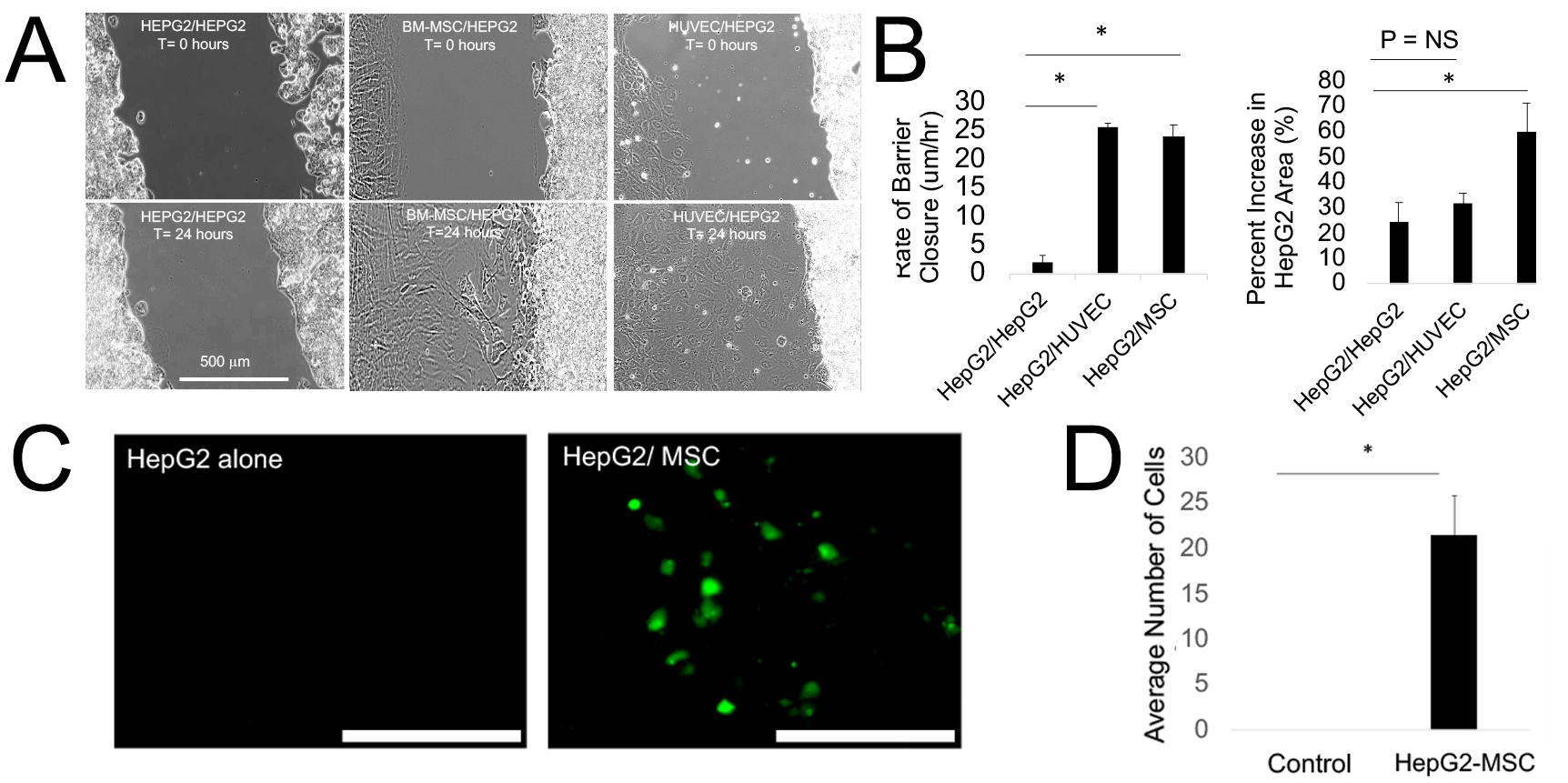

Supplement: Supplementary Figure 1 — (A) Phase-contrast images of 2D barrier migration assays between HepG2 and mesenchymal stem cells (HUVECS and MSCs) on at T = 0 h (top) and T = 24h (bottom). Time lapse performed every 30 min over 24h. (B) Left: bar graph analysis of migration demonstrates a significant increase in barrier closure in the presence of HUVECS (P = 0.00057, n = 3) and hMSCs (P = 0.00028, n = 3) as compared with HepG2 control (n = 3). Right: bar graph analysis of HepG2 border wall movement in the presence of MSCs (P = 0.047, n = 3) as compared with HepG2 control (n = 3). Plotted is mean ± SD. Significance defined as P ≤ 0.05. (C) Fluorescence image focused on a transwell during a transwell assay demonstrating HepG2-GFP migration alone (left) or in the presence of MSCs (right). Bar = 200 μm. (D) Bar graph plotting average number of HepG2-GFP migrating through transwell (P = 0.013, n = 3). Plotted is mean ± SD. Significance defined as P ≤ 0.05. [file Image_1.TIF]

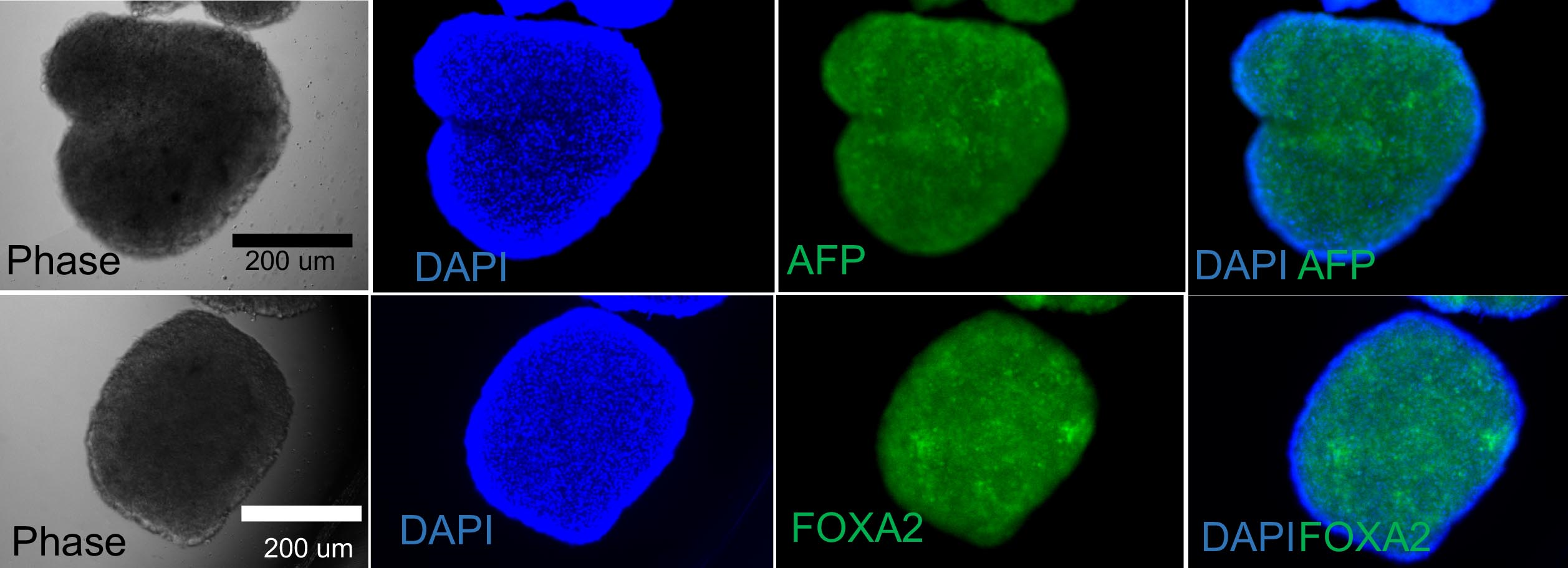

Supplement: Supplementary Figure 2 — HepG2 spheroids in suspension culture were collected and immunolabeled in suspension. Briefly, spheroids were harvested and fixed with 4% paraformaldehyde (PFA) at room temperature, permeabilized with 1% Triton X-100 and then incubated overnight with either alpha-fetoprotein (AFP) or FOXA2 monoclonal primary antibody. These markers are specific to liver lineage commitment. HepG2 spheroids were observed to positively express FOXA2 and AFP. [file Image_2.TIF]

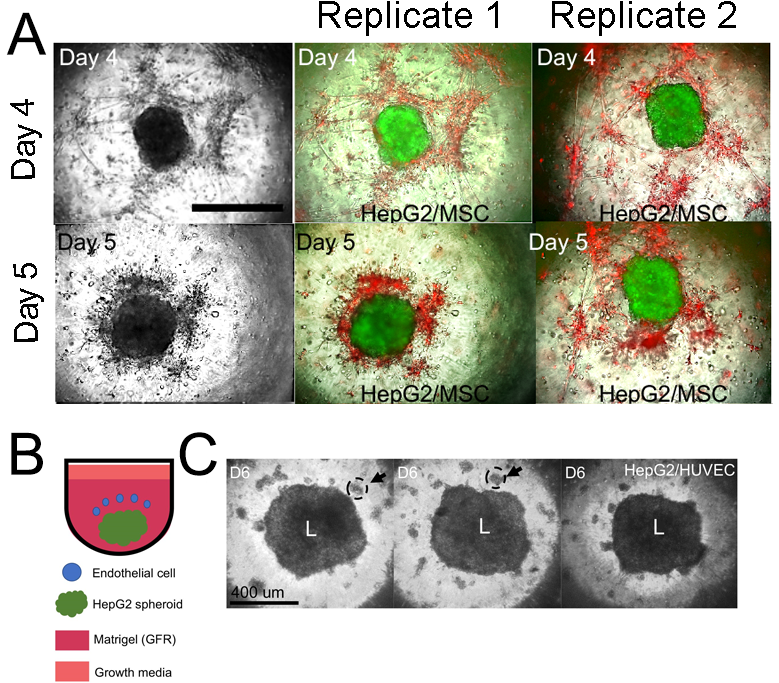

Supplement: Supplementary Figure 3 — (A) HepG2 cells are initially formed into spheroids using 384-well ULA plates over a 3-day culture period. Separately, MSC cells, premixed with MG are collected and seeded as 20,000 cells per microwell containing a HepG2 spheroid. Columns of images are replicates 1 and 2 with double fluorescent images on days 4 and 5. HepG2 (green), MSC (red). Bar = 500 μm. (B) HepG2 spheroids (L) were initially formed in suspension culture using 384-well round-bottom ultra-low attachment plates (1,000 cells per well). On day 3, spheroids were then embedded in MG (GFR) containing 20,000 HUVEC cells. Additional growth media to support HepG2 and endothelial cell proliferation, DMEM:EGM in a 1:1 ratio, was used to culture the embedded spheroids. (C) By day 6, no significant outward migration of HepG2 cells was observed into the ECM. Aggregates of endothelial cells were observed in the MG as clusters (black arrow) that showed no interaction with the HepG2 spheroid. [file Image_3.TIF]

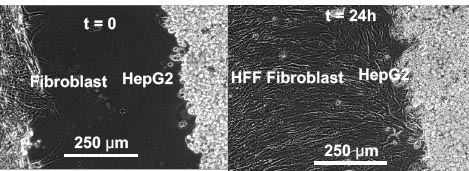

Supplement: Supplementary Figure 4 — A barrier migration assay to establish the effect of human foreskin fibroblasts (HFFs) on HepG2 collective cell migration. Briefly, an IbidiTM two-well insert was used to create a barrier between HFFs and HepG2 cells seeded 500 μm apart. After overnight incubation to allow for cell attachment, the insert was removed to allow for migration between the cell reservoirs. Collective migration of HepG2 cells is observed outwards in response to the presence of the HFFs present. [file Image_4.TIF]

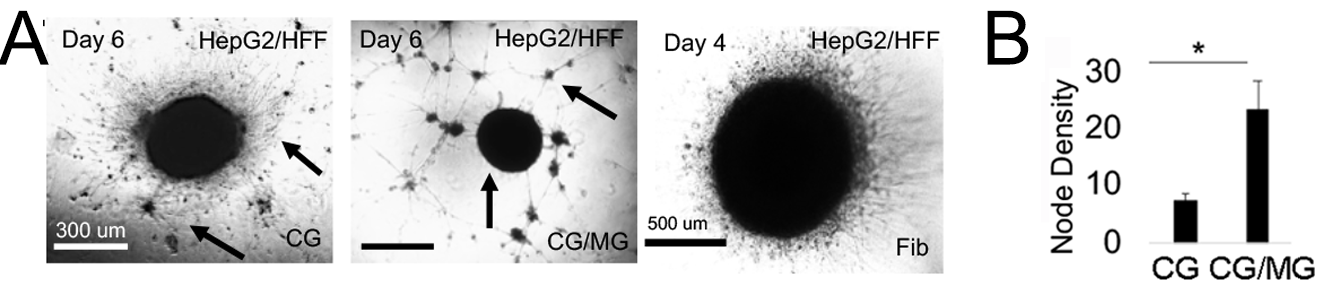

Supplement: Supplementary Figure 5 — (A) Phase-contrast images of HepG2/HFF-mixed spheroids. Left: in collagen gel (CG) on day 6, arrows show hair-like structures migrating. Middle: in CG/MG mixture, arrows show node-like structures in hydrogel. Right: in Fibrin hydrogel, hair-like structures are migrating. (B) Bar graph plotting node density within the mesenchyme, comparing HepG2/HFF spheroids grown in the CG and CG/MG condition (day 6, P = 0.0043, n = 3). Significance defined as P ≤ 0.05. [file Image_5.TIF]

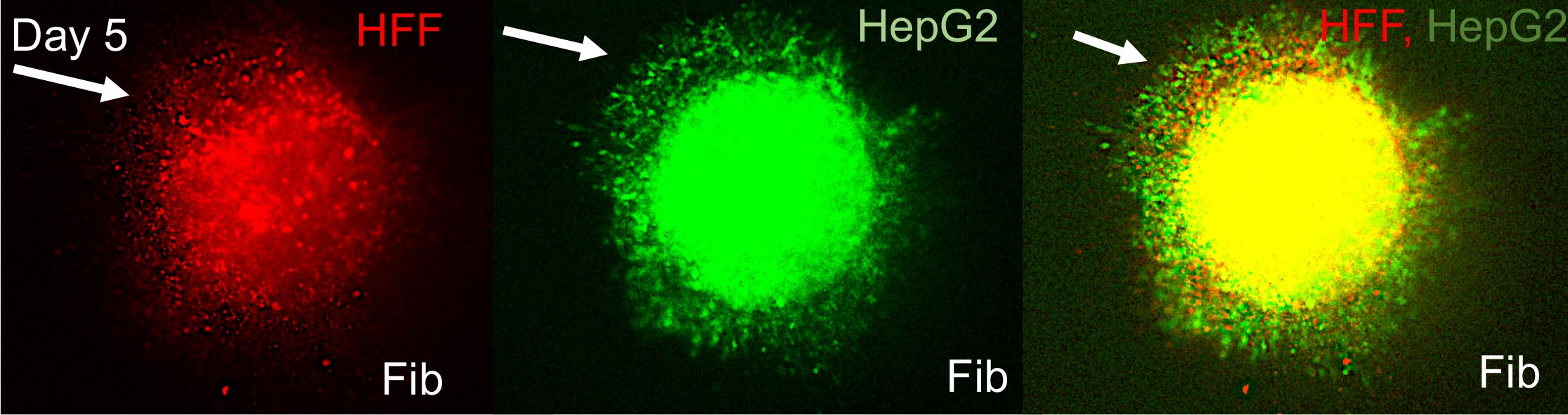

Supplement: Supplementary Figure 6 — HepG2 cells and human foreskin fibroblasts (HFFs) were initially separately dye-labeled green and red, respectively, and then mixed in a 1:1 ratio before being seeded into 384-well round-bottom ultra-low attachment plates. Cells formed a compact spheroid that was subsequently embedded in a premade fibrin hydrogel. Fibrin hydrogel was made by polymerizing fibrinogen (3.25 mg/ml) with thrombin (12.5 U/ml) in a 4:1 ratio. There was enhanced co-migration of HepG2 cells and HFF cells outwards into the fibrin matrix. [file Image_6.TIF]

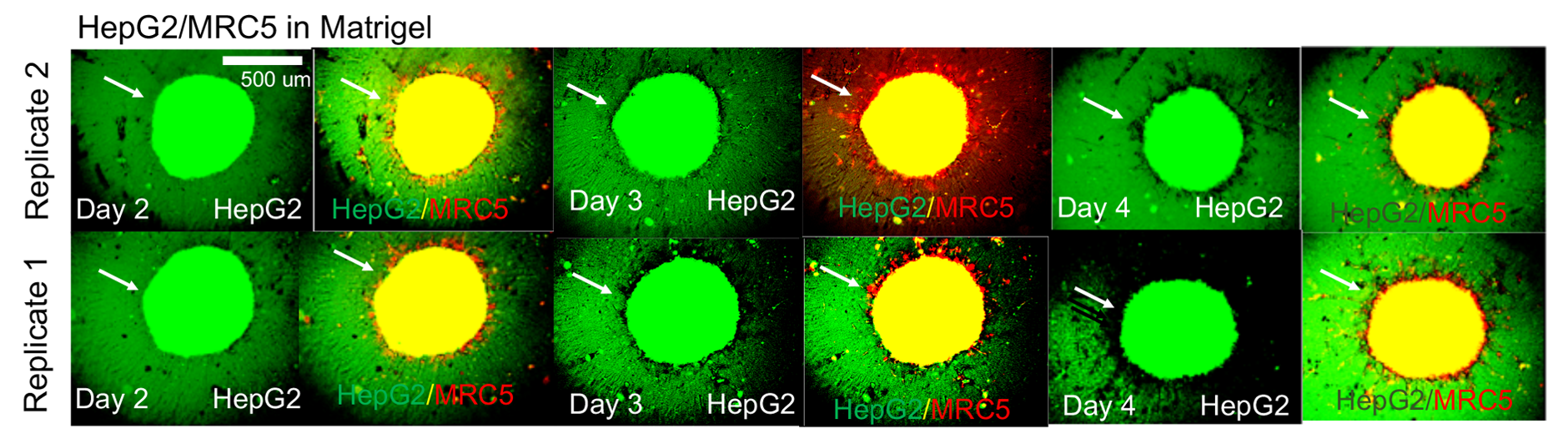

Supplement: Supplementary Figure 7 — Fluorescent images of days 2–4 of HepG2-GFP/MRC-5-mixed spheroids in MG. From left to right: HepG2 (green) cells and combined HepG2 (red) and MRC-5 (yellow) images. Replicates 1 (above) and 2 (below) are shown. Arrows show HepG2 and MRC-5 migration. [file Image_7.TIF]

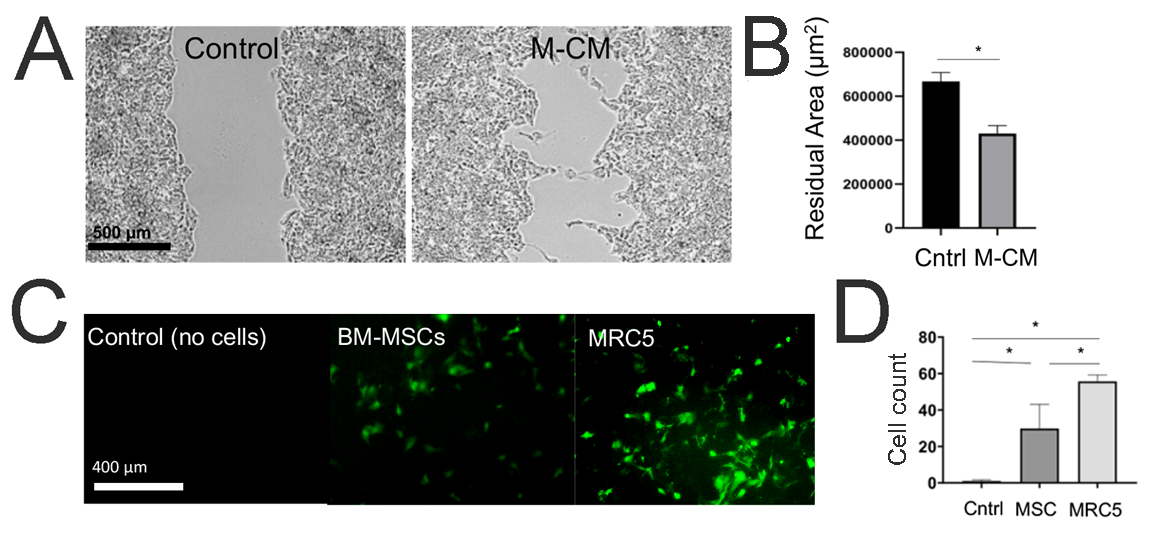

Supplement: Supplementary Figure 8 — MRC-5-conditioned media support liver cell migration in multiple 2D migration assays. (A) Phase-contrast images of 2D barrier migration assays between HepG2 cells (on both sides of the barrier) in the presence of MRC-5-conditioned medium (M-CM) at T = 24h in control (left) and M-CM (right) conditions. (B) Bar graph comparing residual area between control and M-CM conditions (P = 0.00085, n = 3). Plotted is mean ± SD. Significance defined as P ≤ 0.05. (C) Fluorescence image focused on a transwell during a transwell assay demonstrating HepG2-GFP migration alone, or in the presence of MRC-5 (and resulting M-CM), after 24h. (D) Bar graph analyzing transwell assay comparing control with MSC (P = 0.023, n = 3), MSC with MRC-5 MSC (P = 0.012, n = 3), and control with M-CM conditions (P = 0.0014, n = 3). Plotted is mean ± SD. Significance defined as P ≤ 0.05. [file Image_8.TIF]

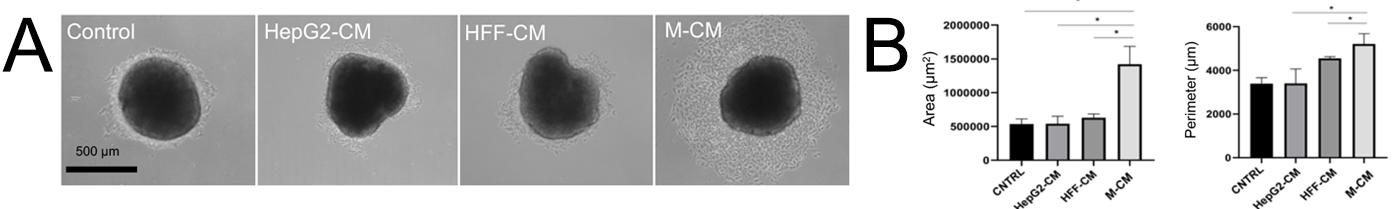

Supplement: Supplementary Figure 9 — Phase-contrast images of HepG2 spheroid outgrowth assay in control, HepG2 CM, HFF-CM, and M-CM (MRC-5 CM). (A) Spheroids cultured on MG (1:15)-coated plates. (B) Bar graph for spheroid outgrowth assay comparing area of spheroid growth (left) and growth perimeter (right) between control, HepG2-CM, HFF-CM, and M-CM conditions. Comparison of spheroid growth area for M-CM and control (P = 0.0023, n = 3), M-CM and HepG2-CM (P = 0.0030, n = 3), and M-CM and HFF-CM (P = 0.0029, n = 3) as shown. Comparison of growth perimeter between M-CM and HepG2-CM (P = 0.0030, n = 3) and M-CM and HFF-CM (P = 0.0029, n = 3) as shown. Plotted is mean ± SD. Significance defined as P ≤ 0.05. [file Image_9.TIF]

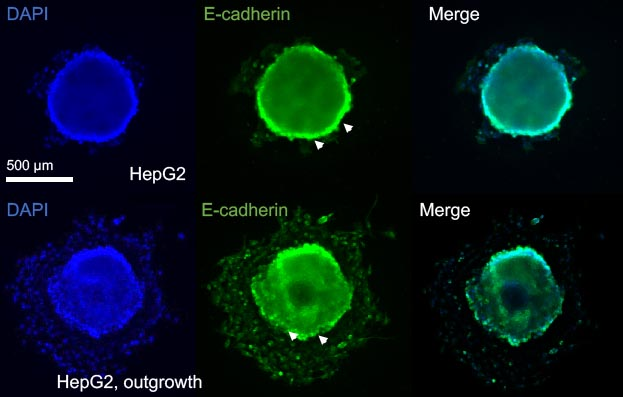

Supplement: Supplementary Figure 10 — Spheroid outgrowth assay in control and M-CM medium with immunocytochemistry for E-cadherin. HepG2 spheroids seeded onto MG-coated plates (1:15 dilution) are allowed to attach (24h). For the top condition, the medium is changed to control medium (DMEM + 10% FBS) and to MRC-5-conditioned media (0.2 μm filtered as normal) for the bottom condition. Attached spheroids are incubated for an additional 24h, and then underwent immunocytochemistry for E-cadherin. Spheroids cultured in MCM showed irregular localization of E-cadherin around the migrating edge of the spheroid, suggestive of collective cell movement outwards. Spheroids cultured in the control condition, exhibited strong expression along the spheroid edge. [file Image_10.TIF]

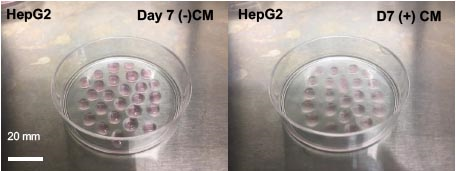

Supplement: Supplementary Figure 11 — Images obtained of day 7 HepG2 MG droplet culture systems in MRC-5-conditioned media (MCM) and control experimental conditions (serum containing DMEM). The MCM condition is a lighter pink color than the control condition which is supportive evidence for the degradation of the MG by the migratory HepG2 cells in response to the presence of the MCM. [file Image_11.TIF]

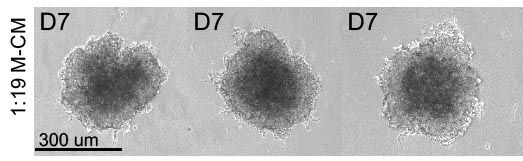

Supplement: Supplementary Figure 12 — HepG2 spheroids cultured in 1:19 diluted MRC-5-conditioned media (M-CM) exhibit no matrix invasion. HepG2 spheroids formed in suspension culture using a 384-well round-bottom ultra-low attachment plate were harvested and seeded into MG droplets on tissue culture-treated 60 mm dishes. MG droplets were then cultured in 1:19 M-CM for an additional 4 days. On day 7, spheroids showed no significant outward migration into the extracellular matrix (ECM). The M-CM media typically supports extensive HepG2 migration. [file Image_12.TIF]

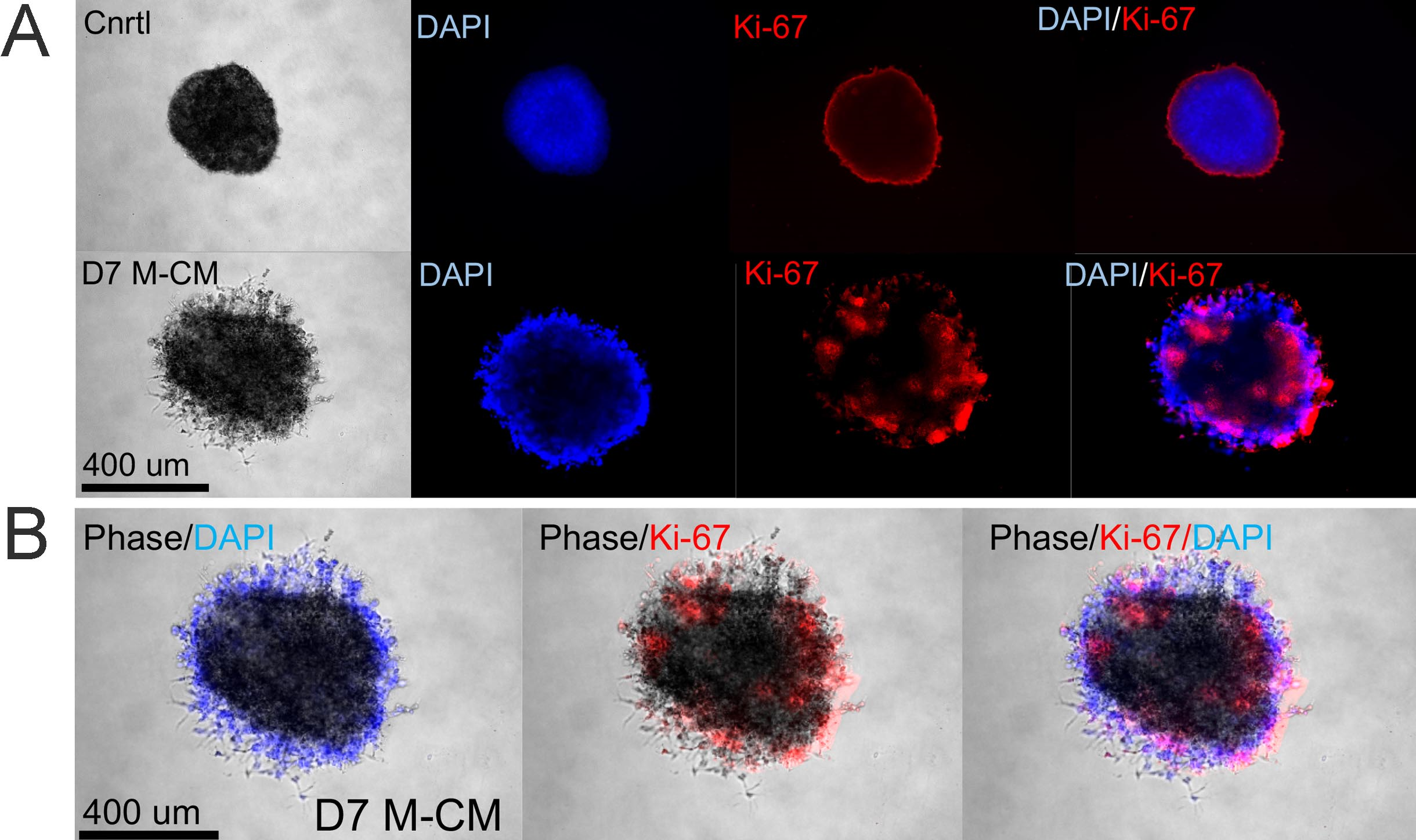

Supplement: Supplementary Figure 13 — Ki67 staining of HepG2 spheroids in MG droplet culture. HepG2 spheroids formed in suspension culture using a 384-well round-bottom ultra-low attachment plate (3-day culture) were harvested and seeded into MG droplets on tissue culture-treated 60 mm dishes. MG droplets were then cultured in MRC-5-conditioned media (M-CM) for an additional 4 days. On day 7, droplets were fixed with 4% paraformaldehyde (PFA) at room temperature, permeabilized with 1% Triton X-100, and stained using monoclonal primary antibody, Ki67, a marker of proliferation. Spheroids treated with M-CM staining showed positive expression for Ki67 localized to migrating cords as compared with control, droplets cultured in serum-containing DMEM. [file Image_13.TIF]

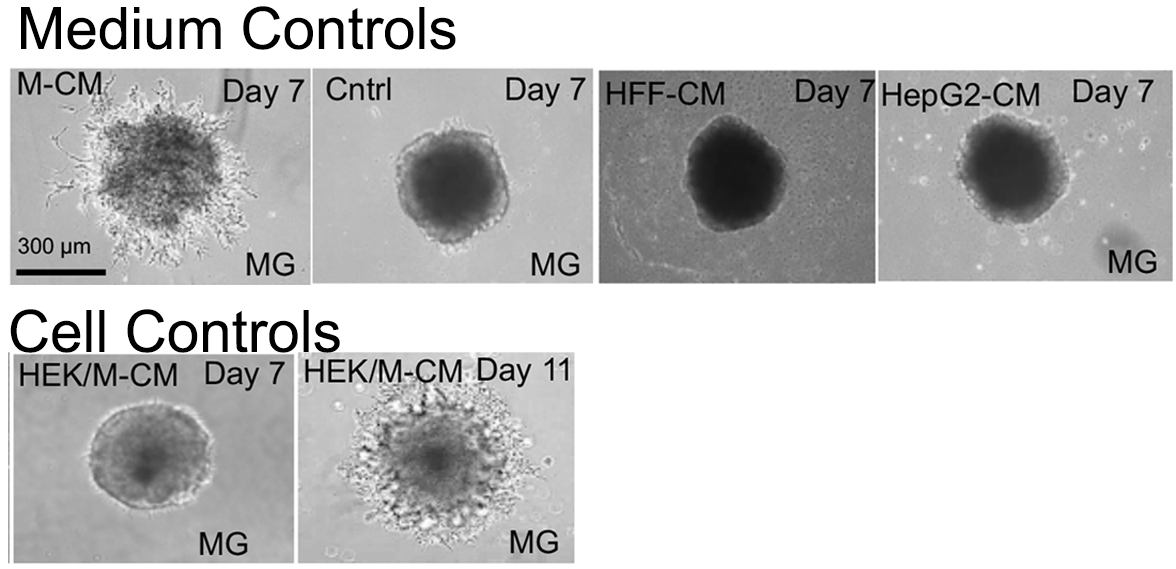

Supplement: Supplementary Figure 14 — Phase-contrast images on day 7 of HepG2 spheroid in MG culture treated with various conditioned medium conditions including M-CM (far left), control (DMEM, 10% FBS, 1% Pen-strep, left), HFF-CM (right), and HepG2-CM (far right). In addition, a separate cell line, human embryonic kidney cells, HEK, were made into spheroids, embedded into MG, and cultured in M-CM (days 7 and 11 are shown). Radial migration is apparent in HepG2 and HEK conditions where M-CM media are included. [file Image_14.TIF]

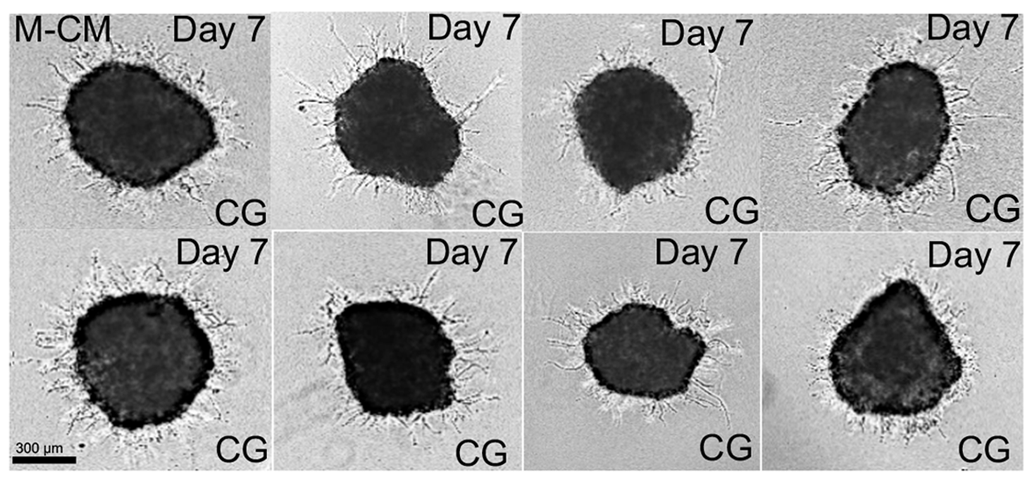

Supplement: Supplementary Figure 15 — Collagen gel-embedded HepG2 spheroids on day 7 exhibit radial migration. HepG2 spheroids were formed in suspension using a 384-well round-bottom ultra-low attachment plate (3-day culture) were collected and seeded into collagen droplets on tissue culture-treated 60 mm dishes. Collagen droplets were then cultured in MRC-5-conditioned media (M-CM) for an additional 4 days. On day 7, migrating spheroids were observed and shown to exhibit thin radial protrusions into the matrix that was consistent among eight individual repeats. [file Image_15.TIF]
